# Gene set analysis approaches for RNA-seq data: performance evaluation and application guideline

**DOI:** 10.1093/bib/bbv069

**Published:** 2015-09-04

**Authors:** Yasir Rahmatallah, Frank Emmert-Streib, Galina Glazko

**Keywords:** RNA-seq, gene set analysis, self-contained, competitive, robustness

## Abstract

Transcriptome sequencing (RNA-seq) is gradually replacing microarrays for high-throughput studies of gene expression. The main challenge of analyzing microarray data is not in finding differentially expressed genes, but in gaining insights into the biological processes underlying phenotypic differences. To interpret experimental results from microarrays, gene set analysis (GSA) has become the method of choice, in particular because it incorporates pre-existing biological knowledge (in a form of functionally related gene sets) into the analysis. Here we provide a brief review of several statistically different GSA approaches (competitive and self-contained) that can be adapted from microarrays practice as well as those specifically designed for RNA-seq. We evaluate their performance (in terms of Type I error rate, power, robustness to the sample size and heterogeneity, as well as the sensitivity to different types of selection biases) on simulated and real RNA-seq data. Not surprisingly, the performance of various GSA approaches depends only on the statistical hypothesis they test and does not depend on whether the test was developed for microarrays or RNA-seq data. Interestingly, we found that competitive methods have lower power as well as robustness to the samples heterogeneity than self-contained methods, leading to poor results reproducibility. We also found that the power of unsupervised competitive methods depends on the balance between up- and down-regulated genes in tested gene sets. These properties of competitive methods have been overlooked before. Our evaluation provides a concise guideline for selecting GSA approaches, best performing under particular experimental settings in the context of RNA-seq.

## Introduction

‘The death of microarrays?’ note, published in 2008 [1], marks the beginning of the ongoing transition from microarrays to whole transcriptome sequencing (RNA-seq). RNA-seq not only enables researcher to identify differentially expressed (DE) genes with higher resolution than microarrays [2], but it also allows to study alternative splicing [3], new coding and noncoding RNA transcripts [4, 5] and long noncoding RNAs [6]. That is, RNA-sequencing answers a much wider range of questions than microarrays. Yet, the basic set of questions asked in regards to RNA-seq data remains the same as before: (Q1) how to identify significantly DE genes with high accuracy; (Q2) how to interpret a long list of seemingly unrelated DE genes; and (Q3) how to gain insights about the biological mechanisms, underlying phenotypic differences, that are not inferable from a list of DE genes.

Microarrays have been used for genome-wide gene expression experiments since 1997 [7], and there are many statistical approaches available for their analysis. Is it possible to apply the same methodologies that were developed for microarrays to answer Q1–Q3 questions for RNA-seq data? Initially the answer was ‘no’. In RNA-seq experiments, the expression level of a transcript is quantified in counts of transcript reads mapped to a genomic region [4, 5]. The read counts are integer numbers, but the methodologies for microarrays model the gene expression by continuous (e.g. normal) distributions. To solve this problem, new models fitting RNA-seq data properties were actively looked for. Specifically, for finding DE genes (Q1), gene counts were modeled using a Poisson or Negative Binomial (NB) distribution and special software packages, such as edgeR [8], DESeq [9] and SamSeq [10], to name a few, were developed. However, recently, a count data transformation was suggested (‘variance modeling at the observational level’, VOOM [11]), and many of the approaches initially developed for finding DE genes for microarrays became applicable for RNA-seq. It was shown that log counts, normalized for sequencing depth and incorporating the mean-variance trend into a precision weight (VOOM procedure), can be entered into the ‘limma’ analysis pipeline, developed for microarrays [12], and then the pipeline performs as well as NB or Poisson methods [11].

The main challenge of analyzing microarray data is not in finding DE genes, but in interpreting the results (i.e. in answering Q2 and Q3 questions). To facilitate interpretability, approaches that incorporate existing biological knowledge (in a form of functionally related gene sets or known biological pathways) into the analysis were developed. The simplest technique incorporating biological knowledge, designed toward interpreting long gene lists (Q2), is the gene set overrepresentation analysis. Here, a set of a priori selected, significantly DE genes, is tested for overrepresentation in annotated gene sets such as Gene Ontology (GO) categories or Kyoto Encyclopedia of genes and genomes (KEGG), using standard statistical tests for enrichment [13]. However, this approach has several shortcomings. First, it does not account for genes with small changes in expression that might be biologically relevant [14] but are almost always absent in the list of statistically significant DE genes. Second, because genes do not work in isolation, statistical tests need to account for the multivariate nature of expression changes [15, 16], but the overrepresentation analysis does not. Notwithstanding the shortcomings, overrepresentation analysis is widely used for microarray data and has been also adapted for RNA-seq data. Specifically, Young and colleagues [17] developed GOseq, a GO categories overrepresentation analysis that accounts for transcript length bias inherent for RNA-seq. The GOANA function in ‘limma’ package is supposed to work similarly to GOseq [18].

The aim of this review is to present an alternative technique that considers differential expression of gene sets and does not require a priori selected genes in the context of RNA-seq data. There are many methodologies developed for microarrays, collectively named gene set analysis (GSA) approaches, which treat a gene set as a unit of expression [16, 19–21]. Overall, the purpose of using GSA is to provide an expansive view of the underlying biological processes, leading to phenotypic differences (Q3 question). The review is organized as follows. In the first part, we overview GSA approaches that can be adapted from microarrays practice to fit RNA-seq data as well as those specifically designed for RNA-seq. While there are plenty of GSA approaches, they are readily distinguished based on the null hypothesis they test. According to Goeman and Buhlmann [22], the formulation can be either ‘self-contained’ or ‘competitive’. ‘Self-contained’ approaches compare whether a gene set is DE between two phenotypes, while ‘competitive’ approaches compare a gene set against its complement that contains all genes except genes in the set [22, 23]. ‘Self-contained’ approaches can be (i) univariate, in a sense that they use gene-level tests for GSA and combine univariate statistics for individual genes into a single test score [19, 24, 25]; and (ii) multivariate, when a multivariate statistic is used to address the null hypothesis. Importantly, gene-level tests for GSA disregard existing correlation structure within a gene set. In real biological settings, moderate [26] and extensive [27] correlations between genes in gene sets are well documented [28] and may result in a decrease of power for gene-level tests as compared with multivariate tests [15, 25, 28, 29]. Hanzelmann *et al*. [30] have suggested to distinguish two groups of ‘competitive’ GSA approaches: (i) ‘supervised’, when the class labels are known; and (ii) ‘unsupervised’, when the enrichment score is computed for each gene set and individual sample. These two terms ‘supervised’ and ‘unsupervised’ are mostly associated with machine learning parlance. For GSA, the ‘supervised’ term simply refers that the samples classification information is known, while the ‘unsupervised’ term indicates that the samples classification is unknown [30], somewhat similar to supervised and unsupervised learning concepts. Another difference is whether the null hypothesis is tested through subject sampling or gene sampling [31]. A number of review articles concerning the different aspects of GSA approaches developed for microarrays data analysis have been published [19, 22, 32–37]. The recommendations expectedly depend on the pool of GSA tests selected for comparisons, biological data sets and simulation strategies used for performance evaluation.

**Figure 1.**
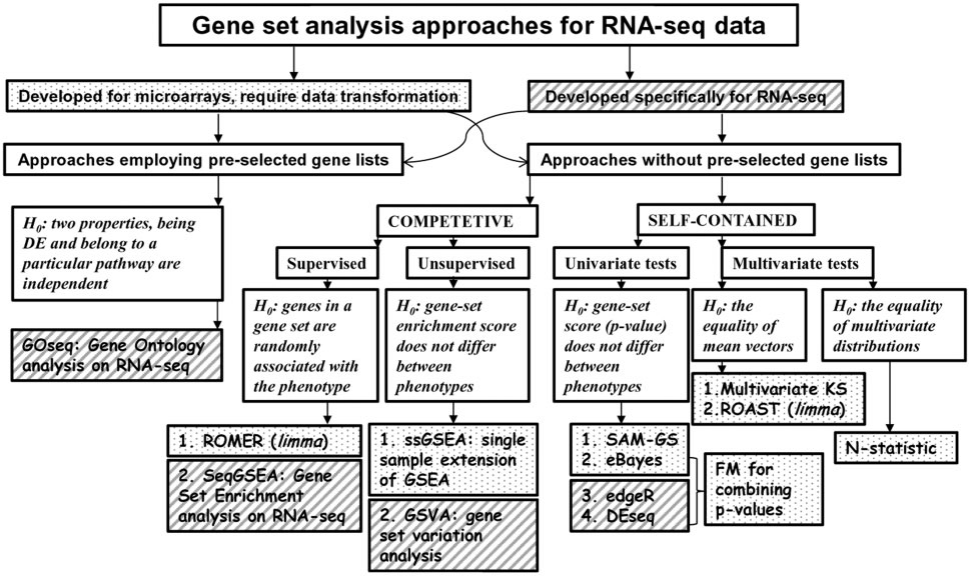
Schematic overview illustrating the breakup of the GSA methods that can be adapted from microarrays practice to fit RNA-seq data (boxes with dots) as well as those specifically designed for RNA-seq (boxes with diagonal stripes) based on the different null hypotheses they test.

In the second part of the review, we attempt to provide a meaningful comparison of the few GSA approaches that cover intrinsically statistically different (in terms of null hypotheses) tests: self-contained (univariate, multivariate) and competitive (supervised, unsupervised). Figure 1 illustrates different null hypotheses tested by different GSA approaches. We assess the performance of different methods in terms of Type I error rate, power and robustness to the sample size and heterogeneity, as well as the sensitivity to different types of selection biases using simulated and real RNA-seq data. Not surprisingly, the performance of various GSA approaches depends only on the statistical hypothesis tested and does not depend on whether the test was developed for microarrays or RNA-seq data. It should be noted that because pathways databases do not include different isoforms of the same gene, GSA approaches operate with genes and not isoforms, with one exception. The SeqGSEA method was proposed specifically with the aim of integrating the differential expression (DE) and differential splicing (DS) analyses from RNA-seq count data with competitive Gene Set Enrichment Analysis (GSEA) [38]. By integrating DE and DS scores, SeqGSEA was able to detect more overrepresented gene sets than without integration [38]. To be as comprehensive as possible, we include SeqGSEA in our analysis.

## Methods

We introduce the following notations. Consider two different biological phenotypes, with *n*_1_ samples of measurements for the first and *n*_2_ samples of the same measurements for the second. Let the two random vectors of *X* = (*X*_1_, … , *X_n_*_1_) and *Y* = (*Y*_1_, … , *Y_n_*_2_) represent the measurements of *p* gene expressions (constituting a pathway) in two phenotypes where *X_i_* corresponds the *i*^th^ *p*-dimensional sample in one phenotype and *Y_i_* corresponds the *i*^th^ *p*-dimensional sample in the other phenotype. Let *X*, *Y* be independent and identically distributed with the distribution functions *F_x_*, *F_y_*, mean vectors *μ_x_* and *μ_y_* and *p* × *p* covariance matrices *S_x_*, *S_y_*.

### *H_0_* for self-contained tests

For multivariate self-contained tests, we consider the problem of testing the general hypothesis *H_0_*: *F_x_* = *F_y_* against an alternative *F_x_* ≠ *F_y_*, or a restricted hypothesis *H_0_*: $\mu_{x}=\mu_{y}$ against an alternative $\mu_{x}\neq \mu_{y}$, depending on a test statistic.

Gene-level GSA approaches test a null hypothesis that the gene-set associated score does not differ between phenotypes. The score can be calculated, for example, as an *L*_2_-norm of the moderated *t*-statistics [39] or as a combined *P*-value [25]. In all cases, statistical significance is evaluated by comparing the observed score with the null distribution, obtained by permuting sample labels.

### *H_0_* for competitive tests

Barry *et al*. [31] have introduced the statistical framework for classifying null hypotheses that are tested by different competitive GSA approaches. In their framework, all competitive tests belong to either ‘Class 1’ or ‘Class 2’ gene category tests. The major distinction is that to evaluate significance of the global test statistic, Class 1 approaches use gene sampling, while Class 2 approaches use subject sampling [31]. The first competitive GSA test for microarray data analysis, GSEA method [14, 40], belongs to Class 2. As a local test statistic, it uses a signal to noise ratio and a weighted Kolmogorov–Smirnov (KS) as a global test statistic (enrichment score, normalized to factor out the gene set size dependence) [34, 40]. Assuming a null distribution $F_{0}{}^{perm}$ induced by permuting sample labels, GSEA evaluates significance of the global test statistic $\zeta_{k}{}^{GSEA}$ by estimating nominal *P*-value from $F_{0}{}^{perm}$ [34, 40]. Thus, GSEA tests the null hypothesis that the genes in a gene set are randomly associated with the phenotype.

Most competitive GSA approaches are ‘supervised’, in a sense that sample labels are known (that is, there are at least two different phenotypes). Hazelmann *et al*. [30] formulated the concept of ‘unsupervised’ GSEA where an enrichment score is computed for each gene set and individual sample [30]. Essentially, unsupervised competitive GSA approach implements a ‘dimensionality reduction’ by transforming a matrix of gene expressions across samples into a matrix of gene sets enrichment scores across the same samples. It makes the choice of null hypothesis flexible and context dependent. For example, Barbie *et al*. [41] use unsupervised competitive GSEA to test the null hypothesis that the Spearman correlation between gene set enrichment scores is zero, while Hazelmann *et al*. [30] test the hypothesis that gene set enrichment score does not differ between two phenotypes.

## RNA-SEQ counts normalization

Raw RNA-seq counts are neither directly comparable between genes within one sample nor between samples for the same gene. Longer genes produce more reads in the sequencing process; therefore, the counts of each gene are proportional to both gene abundance and gene length. The counts will also vary between samples as a result of differences in the total number of mapped counts per sample (library size or sequencing depth). The first normalization for RNA-seq data ‘reads per kilobase per million’ (RPKM) was suggested by Mortazavi *et al*. [42]. While RPKM remains popular, a number of other normalizations were suggested [9, 43–45]. Recently, we have shown that in the context of multivariate self-contained GSA approaches Type I error rate and power were severely affected by different test statistics but virtually unaffected by the normalization used [25]. Therefore, in what follows, we use RPKM for multivariate self-contained GSA approaches. VOOM normalization is used for all GSA approaches initially developed for microarrays. Gene-level GSA as well as competitive GSA approaches developed specifically for RNA-seq are used with the approach-specific normalization (for more detailed description of the normalization methods see Supplementary File 1).

## Self-contained gene-level tests for GSA

### Gene-level GSA tests that combine genes *P*-values

One way of designing a GSA test is to combine univariate statistics for individual genes into a single test score [19, 24]. There are two popular univariate tests specifically developed for RNA-seq data that rely on NB model for read counts: edgeR [8] and DESeq [9]. Both tests do not require RNA-seq counts normalization and adjust only for differing library sizes between samples automatically. In the context of microarray data, Empirical Bayes method (eBayes [12]) correctly identifies hypervariable genes and can be adapted for RNA-seq data through VOOM normalization [11]. In what follows, we briefly reiterate the conclusions from our comparative power and Type I rate analyses of different gene-level GSA tests [25]. Our major conclusion was that, when applied correctly, the gene-level test does not, *per se*, influence the performance of a gene-level GSA approach as much as the procedure used to combine univariate statistics into a single test score does [25].

The first gene-level GSA approach for RNA-seq data was suggested by Fridley *et al*. [46]. As a gene-level test, the authors [46] selected edgeR. For every gene set, edgeR-generated genes’ *P*-values were combined into a single test *P*-value using Gamma Method (GM) [46]. GM is based on summing the transformed gene-level *P*-values using an inverse gamma cumulative distribution function. The statistical significance of the combined *P*-values was estimated from the null distribution obtained by subject sampling [46]. There are many other well-known methods to combine *P*-values, e.g. the Fisher [47] or Stouffer [48] methods (FM and SM in what follows). We have shown that gene-level GSA tests that use GM for combining *P*-values had the highest power and Type I error rate on simulated and real data [25]. In turn, tests with SM had the smallest power and the smallest Type I error rates, while the results for tests with FM were intermediate [25]. If one would like to design a gene-level GSA test for RNA-seq data and combine test-generated *P*-values into a single gene set *P*-value, the safest option would be to use FM. In this review, gene-level tests for GSA are represented by edgeR, DESeq and eBayes in combination with FM (for more detailed description of the methods for combining *P*-values see Supplementary File 1).

### Gene-level GSA test that combines statistics (SAM-GS)

In the analysis of microarrays, shrinking the standard error of a test statistic (e.g. a *t*-test) in testing DE of individual genes improves the power of the test. Several shrinkage approaches at the level of individual genes were suggested, including the Significance Analysis of Microarrays (SAM) test [49], the regularized *t*-test [50] and the moderated *t*-test [51]. In particular, an extension of SAM test to GSA (SAM-GS) was suggested [39] and has been demonstrated to outperform several conventional self-contained tests and even the original competitive GSEA approach [21, 39, 52].

SAM-GS can be applied to RNA-seq count data by using the VOOM normalization [11] before the test to find the log-scale counts per million (CPM) of the raw counts normalized for library sizes. The test statistic is the *L*_2_-norm of the moderated *t*-statistics for the gene expressions:

$$T_{SAM-GS}=\overset{p}{\underset{i=1}{\sum }}{\left(\frac{{\overset{¯}{X}}_{i}-{\overset{¯}{Y}}_{i}}{s_{i}+s_{0}}\right)}^{2}$$

where ${\overset{¯}{X}}_{i}$ and ${\overset{¯}{Y}}_{i}$ are respectively the mean expression levels for gene *i* under phenotypes *X* and *Y*, *s_i_* is a pooled standard deviation over the samples in the two phenotype, *s*_0_ is a small positive constant to adjust for small variability and *p* is the number of genes in the gene set.

## Self-contained multivariate tests for GSA

### Multivariate generalization of the KS test

The multivariate generalization of the KS test suggested by Friedman and Rafsky [53] that we adapted for GSA [24] is based on the Minimum Spanning Tree (MST) ranking. The multivariate generalization of KS ranks multivariate observations based on their MST. The purpose of MST ranking is to obtain the strong relation between observation differences in ranks and their distances in *R^p^*. Multivariate KS tests the hypothesis that there is no difference in mean vectors for a gene set between two phenotypes (*H_0_*: $\mu_{x}=\mu_{y}$) [53] (for more detailed description see Supplementary File 1).

### N-statistic

Based on their high power and popularity, we consider two other multivariate test statistics. N-statistic [54, 55] tests the most general hypothesis *H*: *F_x_* = *F_y_* against a two-sided alternative *F_x_* ≠ *F_y_*:

$$N_{n_{1}n_{2}}=\frac{n_{1}n_{2}}{n_{1}+n_{2}}{\left[\begin{array}{l} \frac{1}{n_{1}n_{2}}\overset{n_{1}}{\underset{i=1}{\sum }}\overset{n_{2}}{\underset{j=1}{\sum }}L\left(X_{i},Y_{j}\right)-\frac{1}{2n_{1}^{2}}\overset{n_{1}}{\underset{i=1}{\sum }}\overset{n_{2}}{\underset{j=1}{\sum }}L\left(X_{i},X_{j}\right)\\ -\frac{1}{2n_{2}^{2}}\overset{n_{1}}{\underset{i=1}{\sum }}\overset{n_{2}}{\underset{j=1}{\sum }}L\left(Y_{i},Y_{j}\right) \end{array}\right]}^{1/2}$$

Here we consider only L(X,Y)=X−Y, the Euclidian distance in *R^p^*.

### ROAST

In the context of microarray data, a parametric multivariate rotation gene set test (ROAST) has become popular for the self-contained GSA approaches [56]. ROAST uses the framework of linear models and tests whether for all genes in a set, a particular contrast of the coefficients is nonzero [56]. It can account for correlations between genes and has the flexibility of using different alternative hypotheses, testing whether the direction of changes for a gene in a set is ‘up’, ‘down’ or ‘mixed’ (up or down) [56]. For all comparisons implemented here, the ‘mixed’ hypothesis was selected. Using ROAST with RNA-seq count data requires proper normalization. The VOOM normalization [11] was proposed specifically for this purpose where log CPM, normalized for library size are used. In addition to counts normalization, VOOM calculates associated precision weights, which can be incorporated into the linear modeling process within ROAST to eliminate the mean-variance trend in the normalized counts [11].

## Supervised competitive tests for GSA

### ROMER

The first competitive GSA test for microarray data analysis (GSEA [14]) was developed a decade ago. The original GSEA was sensitive to the gene set size and the influence of other gene sets [57], so it was subsequently upgraded into GSEA-P that used a correlation-weighted KS statistic, an improved enrichment normalization and an FDR-based estimate of significance [34, 40]. For the sake of simplicity, we will only consider the GSEA version implemented in ‘limma’ function ROMER (the rotation testing using mean ranks) [18]. ROMER is a parametric method developed originally for microarray data and uses the framework of linear models [11] and rotations instead of permutations (see [56] for more detail). In contrast to ROAST, the ‘limma’ implementation of ROMER does not incorporate the weights estimated by VOOM into the linear modeling process to account for the mean-variance trend in the data.

### SeqGSEA

The SeqGSEA method was proposed with the aim of integrating the DE and DS analyses from RNA-seq count data with competitive GSEA [38]. The analysis of DE is divided into two parts: DE analysis using gene read counts and DS analysis based on sub-exon counts. Sub-exon counts are defined as nonoverlapping exon fragments. The read counts are modeled using NB distribution. Gene read counts are defined by summing up the read counts of all sub-exons in a gene. DE scores are defined from gene read counts and DS scores are defined from sub-exon read counts as an average value across all sub-exon in a gene [38]. After the estimation of DE and DS scores, they can be combined together into an integrated gene score (using linear combination or rank-based strategy) that reflects the abundance differences between two phenotypes [38]. However, because we are interested in DE analysis only, we followed the exemplified pipeline for such analysis as suggested in the Bioconductor *SeqGSEA* package vignette [58].

## Unsupervised competetive tests for GSA

The goal of unsupervised competitive approaches is to characterize the degree of expression enrichment of a gene set in each sample within a given data set [41]. The term ‘competitive’ is reminiscent of the way the enrichment score is calculated: as a function of gene expression inside and outside the gene set.

### Gene set variation analysis

Gene set variation analysis (GSVA) can be applied to microarray expression values or RNA-seq counts. Depending on the data type, expression values (counts) are first transformed using a Gaussian (or discrete Poisson) kernel into expression-level statistics [30]. The sample-wise enrichment score for a gene set is calculated using KS like random walk statistic. An enrichment statistic (GSVA score) can be calculated as its maximum deviation from zero over all genes (similar to the original GSEA) or as the difference between the largest positive and negative deviations from zero (see [30] for more details).

### Single sample extension of GSEA

The difference between GSVA and single sample extension of GSEA (ssGSEA) stems from the way an enrichment score is calculated. In ssGSEA the enrichment score for a gene set under one sample is calculated as a sum of the differences between two weighted empirical cumulative distribution functions of gene expressions inside and outside the set [41]. The approach, together with GSVA, is implemented in the Bioconductor *GSVA* package [30].

## Simulated and real data sets

### Nigerian data set

To evaluate the performance of different approaches on real data, we used a subset of the Pickrell *et al*. [59] data set of sequenced cDNA libraries generated from 69 lymphoblastoid cell lines that were derived from Yoruban Nigerian individuals. The Nigerian data set was selected for its balanced samples and adequate sample size. Among available samples, only 58 unrelated individuals were considered (29 males and 29 females) (a more detailed description of the preprocessing steps of the Nigerian data set is provided in Supplementary File 1).

### Simulation setup

Simulated data were used to estimate Type I error rate and power. We model the count for gene *i* in sample *j* by a random variable $Y_{ij}$ with NB distribution

$$Y_{ij}∼NB\left(mean=\mu_{ij},\;var=\mu_{ij}(1+\mu_{ij}\varphi_{ij})\right)=NB\left(\mu_{ij},\varphi_{ij}\right)$$

where $\mu_{ij}$ and $\varphi_{ij}$ are respectively the mean count and dispersion parameter of gene *i* in sample *j*. For each gene in a gene set, a vector of mean count, dispersion and gene length information $\left(\mu_{i},\varphi_{i},L_{i}\right)$ is randomly picked from a pool of vectors derived from the processed Nigerian data set. Dispersion parameters for individual genes were estimated using the Bioconductor *edgeR* package [8] (for more detailed description of the simulation setup see Supplementary File 1).

### Molecular signature database

We used the C2 group of gene sets from the molecular signature database (MSigDB) 4.0 [60]. These gene sets (4722) were collected from various sources such as online pathway databases (KEGG, GO, Biocarta, Reactome), publications in PubMed and expert knowledge. The list of gene sets was downloaded and accessed in R using the Bioconductor *GSEABase* package.

## Performance evaluation

### Type I error rate (simulated data)

Random counts following the NB distribution were generated using the pool of parameter vectors obtained earlier from the Nigerian data set. To simulate the null hypothesis *H_0_*: F=G, we generated a data set consisting of *N* samples (equally separated into two phenotypes) and 1000 gene sets of equal size (*p*). Hence, we have a data set of *N* samples and 1000 × *p* genes. The randomly selected parameter vector $\left(\mu_{i},\varphi_{i},L_{i}\right)$ is used to generate NB counts for gene *i* for all the samples in the data set. To examine the effects of sample size and gene set size, we estimated the Type I error rate under different parameter settings for all statistical methods. We chose *p*∈{16, 60, 100} and *N*∈{10, 20, 40, 60}. Type I error rate for a statistical test is calculated as the proportion of gene sets detected by the test. The results were averaged over 10 generated data sets to obtain more stable results.

### Power (simulated data)

In real data, DE gene set may include genes that are up-regulated, down-regulated, similarly expressed between two phenotypes, with variable fold change. In addition, competitive GSA approaches study the enrichment of a gene set in a large number of genes that form multiple gene sets. Other gene sets may influence the power of competitive tests to some (unknown) degree. Therefore, to mimic real data as closely as possible, three simulation parameters were introduced: *β*, the proportion of gene sets in the data set that have truly DE genes; *γ*, the percentage of genes, truly DE in each gene set and *FC*, the fold change in gene counts between two phenotypes. We consider *β* ∈ {0.05, 0.25} and *γ* ∈ {0.125, 0.25, 0.5}. For the parameter *FC*, the values are in the range [1.2, 3]. To represent two biological conditions with different outcomes, two groups with equal sample size, *N*/2 (*N* = 20 and *N* = 40), were considered. For each group, *S* = 1000 nonoverlapping gene sets, each constructed from *p* random realizations of NB distribution, were formed. Relatively small (*p* = 16) and large (*p* = 100) gene set sizes were chosen. The power for all methods was estimated by testing the hypothesis *H_0_*: $\mu_{x}=\mu_{y}$ (or *H_0_*: FC=1) against an alternative *H*_1_: $\mu_{x}\neq \mu_{y}$ (or *H*_1_: FC≠1) for all gene sets. For each of the (1-*β*)*S* non-DE gene sets, *p* random realizations of $NB(\mu_{i},\varphi_{i})$ were sampled, where 1 ≤ *i* ≤ *p* under both phenotypes. For each of the *βS* gene sets that have truly DE genes, *p*/2 random realizations of $NB(\mu_{i},\varphi_{i})$ and $NB(FC\;\mu_{i},\varphi_{i})$ were sampled, under phenotype 1 and phenotype 2 for 1 ≤ *i* ≤ *γp*/2. Also *p*/2 random realizations of $NB(FC\;\mu_{i},\varphi_{i})$ and $NB(\mu_{i},\varphi_{i})$ were sampled under phenotype 1 and phenotype 2 for (*γp*/2) + 1 ≤ *i* ≤ *γp*. In this way, half of the *γp* DE genes in each gene set were up-regulated and half were down-regulated between the two phenotypes.

### Robustness to samples size and heterogeneity (the Nigerian data set)

Because there is no ‘gold standard’ set of pathways that are truly DE (or significantly enriched) for male and female samples supported with high experimental evidence, we created a ‘surrogate’ gold standard set using the procedure suggested in [28, 37] and the full data set (*N* = 58). A total of 3890 C2 gene sets containing 11 903 unique annotated genes in 58 samples (29 males and 29 females) were analyzed. Gene sets detected using the full data set are considered ‘true positives’ *GS_TP_*(*N*) (at the significance level *α* = 0.05) and undetected sets are ‘true negatives’, *GS_TN_*(*N*). For four different sample sizes, *n* = {48, 38, 28, 18}, *B* = 100 subsets were constructed by subsampling without replacement. Each subset consists of two balanced parts that were obtained by subsampling without replacement from male and female samples separately. All statistical methods were applied to these subsets, and the detected C2 gene sets were compared with *GS_TP_*(*N*) and *GS_TN_*(*N*). The rate at which a statistical method detects gene set *i* from the list *GS_TP_*(*N*) in *B* subsets with sample size *n* and a statistical significance level *α* was estimated as

$$m\left(i,n\right)=\frac{1}{B}\overset{B}{\underset{j=1}{\sum }}I\left[P_{i}^{j}(n)<\alpha \;|\;i\in GS_{TP}(N)\right]$$

where $P_{i}^{j}(n)$ is the estimated *P*-value for gene set *i* from the list *GS_TP_*(*N*) in subset *j* with sample size *n*. To find the true-positive rate (TPR) of detecting arbitrary gene sets from list *GS_TP_*(*N*) when subsets of the full data set with sample size *n* were used, *m*(*i*,*n*) was averaged over all members of *GS_TP_*(*N*)

$$TPR\left(n\right)=\frac{1}{\left|GS_{TP}(N)\right|}\overset{\left|GS_{TP}(N)\right|}{\underset{i=1}{\sum }}m(i,n)$$

This measure provides an estimate for the probability to detect gene sets from list *GS_TP_*(*N*) when subsets of sample size *n* are used, which correspond to the power or sensitivity of the statistical method. Similarly, the false-positive rate (FPR) or Type I error rate of a statistical method in detecting gene set *i* from the list *GS_TN_*(*N*) in *B* subsets with sample size *n* and a statistical significance level *α* was estimated as

$$e\left(i,n\right)=\frac{1}{B}\overset{B}{\underset{j=1}{\sum }}I\left[P_{i}^{j}(n)<\alpha \;|\;i\in GS_{TN}(N)\right]$$

and the FPR of detecting arbitrary gene sets from list *GS_TN_*(*N*) when subsets of sample size *n* were used was averaged over all members of *GS_TN_*(*N*):

$$FPR\left(n\right)=\frac{1}{\left|GS_{TN}(N)\right|}\overset{\left|GS_{TN}(N)\right|}{\underset{i=1}{\sum }}e(i,n)$$

Owing to the fact that the lists *GS_TP_*(*N*) and *GS_TN_*(*N*) do not necessarily contain true references, the estimated TPR and FPR here assess the robustness of the methods with respect to the sample size rather than representing true values for the methods [61].

**Figure 2.**
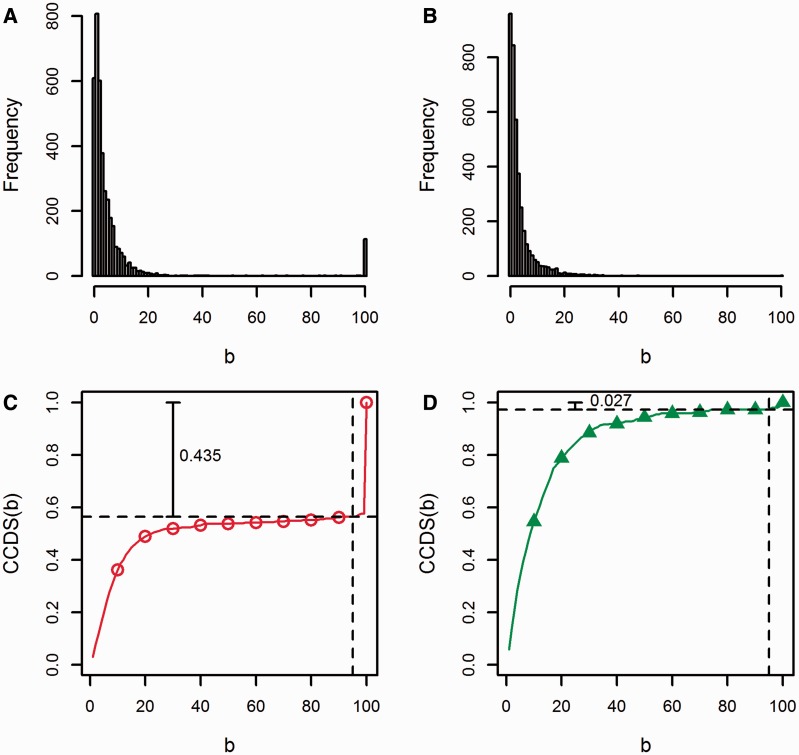
Illustrative histograms and corresponding CCDS curves obtained using commonly detected C2 gene sets at a significance level of 0.05 in 100 subsets of the Nigerian data set with sample size 28. (**A**) Histogram of the number of commonly detected C2 gene sets by N-statistic in *b* subsets out of 100; (**B**) histogram of the number of commonly detected C2 gene sets by GSVA in *b* subsets out of 100; (**C**) CCDS curve showing the CCDS for N-statistic; (**D**) CCDS curve showing the CCDS for GSVA.

To examine the robustness of different GSA approaches to samples heterogeneity, we constructed *B* = 100 subsets for four different sample sizes *n* = {48, 38, 28, 18} and cumulatively quantified the proportion of common gene sets detected in *b* or less subsets (*b* ∈ [1,*B*]). First, each detected gene set could be detected in *b* subsets out of all *B* subsets. The entire range [1,*B*] was divided into *B* bins and we calculated the count in bin *k* (*s_k_*) as the number of gene sets commonly detected in *k* subsets. The resulting bins formed a histogram that illustrated the frequency of commonly detecting gene sets in subsets. Second, we defined the cumulative common detection per subset (CCDS) as the proportion of gene sets commonly detected in *b* or less subsets out of a total of *B* subsets

$$CCDS\left(b\right)=\frac{1}{Q}\;\overset{b}{\underset{k=1}{\sum }}k\;s_{k}$$

where *Q* is the sum of the numbers of detected gene sets in all *B* subsets and *b* = 1, 2, … , *B*. Normalizing by the method-specific *Q* scales the CCDS values of all methods between 0 and 1. Plotting CCDS(*b*) versus *b* provided comparable nondecreasing curves, which assess the robustness of different methods to samples heterogeneity between subsets. To illustrate this approach, Figure 2 shows the histograms and the corresponding CCDS curves obtained by following our procedure for the detected C2 gene sets by N-statistic (Figure 2A and C) and GSVA (Figure 2B and D) in 100 subsets of the Nigerian data set (with sample size 28). Owing to samples heterogeneity between subsets, many gene sets were commonly detected in only few subsets. This corresponds to the rapid rise in CCDS(*b*) at low *b* (Figure 2A and B). However, while 114 gene sets were commonly detected in all 100 subsets by N-statistic (Figure 2A), only 2 were detected by GSVA (Figure 2B). In fact, among the 26 844 gene sets detected by N-statistic in all 100 subsets, 43.5% of them were commonly detected in 95% of all subsets (Figure 2C). This ratio falls drastically to 2.7% of the 14 492 detected gene sets by GSVA in all 100 subsets (Figure 2D). This example demonstrates that N-statistic is more robust to samples heterogeneity, as compared with GSVA. Also, it exemplifies the basis for comparing the CCDS(*b*) curve patterns: (i) for a method robust to samples heterogeneity, CCDS(*b*) is relatively low for small *b* and shows an abrupt rapid rise at high values of *b* (rise owing to gene sets commonly detected in most subsets); (ii) for a method sensitive to samples heterogeneity, CCDS(*b*) increases rapidly and flattens at low *b*, indicating that no gene sets were commonly detected in the majority of subsets. The CCDS curves for different statistical methods using subsets of the Nigerian data set with different sample sizes are presented in the Results Section.

## Results

### Type I error rate (simulated data)

Table 1 presents the estimates of the attained significant levels for all GSA tests considered (*α* = 0.05). Overall, multivariate self-contained tests control Type I rather well except KS (high Type I error rate for small sample size). For gene-level GSA approaches, where *P*-values are combined using FM, edgeR shows the highest Type I error, followed by DESeq and eBayes. The smallest sample size (five by five groups) does not influence Type I error rate in the case of eBayes, but edgeR and DESeq Type I error rates are affected. This could be attributed to eBayes’s conservative empirical approach, which shrinks sample variance estimates toward a pooled estimate, resulting in more stable inference when the number of samples is small [61]. All competitive GSA approaches provide Type I error rates estimates near nominal *α* = 0.05, except SeqGSEA where Type I error rate increases with the sample size (see the Discussion section for a plausible explanation).

**Table 1.** Type I error rates for different methods,*α*  = 0.05

*Method placement*		Self.			*N-statistic*		*SAM-GS*		*KS*		*ROAST*		
		Comp.			*SeqGSEA*		*GSVA*		*ssGSEA*		*ROMER*		
		Comb.			*edgeR_FM*		*DESeq_FM*		*eBayes_FM*		*-*		
		
		*p* = 16				*p* = 60				*p* = 100			
*N*=10	Self.	0.049	0.044	0.084	0.043	0.048	0.045	0.081	0.042	0.048	0.045	0.081	0.041
	Comp.	0.025	0.042	0.042	0.047	0.017	0.047	0.050	0.050	0.013	0.045	0.046	0.047
	Comb.	0.088	0.077	0.047	–	0.127	0.111	0.042	–	0.159	0.137	0.044	–

*N* = 20	Self.	0.052	0.046	0.090	0.044	0.055	0.050	0.090	0.047	0.051	0.055	0.086	0.050
	Comp.	0.040	0.047	0.045	0.051	0.038	0.041	0.047	0.054	0.037	0.050	0.050	0.053
	Comb.	0.072	0.063	0.048	–	0.100	0.079	0.051	–	0.114	0.083	0.054	–

*N* = 40	Self.	0.054	0.054	0.070	0.051	0.047	0.047	0.066	0.044	0.050	0.053	0.068	0.055
	Comp.	0.051	0.044	0.051	0.050	0.057	0.048	0.046	0.045	0.060	0.049	0.053	0.052
	Comb.	0.066	0.058	0.051	–	0.077	0.062	0.047	–	0.088	0.068	0.055	–

*N* = 60	Self.	0.051	0.051	0.058	0.052	0.046	0.047	0.054	0.048	0.049	0.054	0.059	0.054
	Comp.	0.060	0.046	0.051	0.051	0.061	0.051	0.045	0.049	0.066	0.047	0.045	0.050
	Comb.	0.065	0.055	0.052	–	0.063	0.056	0.046	–	0.079	0.065	0.055	–

### Power (simulated data)

**Figure 3.**
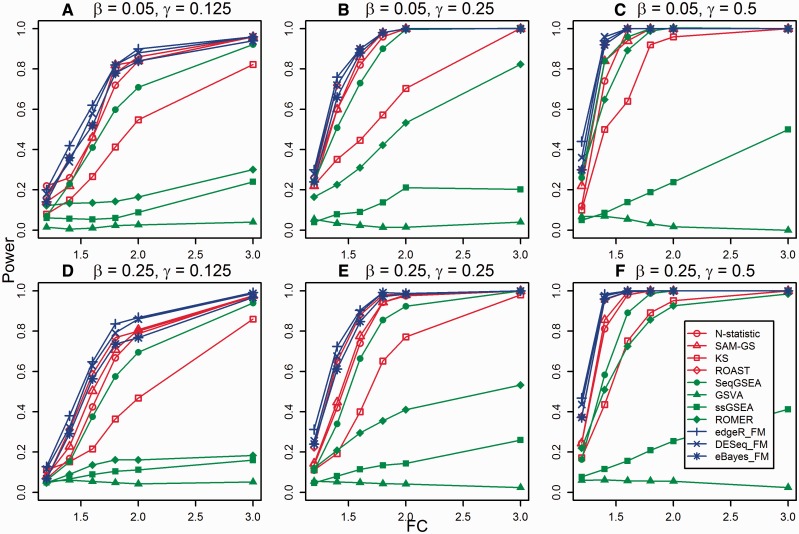
The power of different tests to detect differences between two groups of samples when the alternative hypothesis (*H_1_*) holds true with different settings (values of *β*, *γ* and *FC*). The gene set size is *p* = 16 and the sample size in each group is *N*/2 (*N* = 20). (A) β = 0.05, γ = 0.125; (B) β = 0.05, γ = 0.25; (C) β = 0.05, γ = 0.5; (D) β = 0.25, γ = 0.125; (E) β = 0.25, γ = 0.25; (F) β = 0.25, γ = 0.5. A colour version of this figure is available at BIB online: http://bib.oxfordjournals.org.

Figure 3 presents the power estimates when *H*_1_: $\mu_{x}\neq \mu_{y}$ is true (*N* = 20, *p* = 16). The power estimates for *N* = 20, *p* = 100 (Supplementary File 2: Figure S1), *N* = 40, *p* = 16 (Supplementary File 2: Figure S2) and *N* = 40, *p* = 100 (Supplementary File 2: Figure S3) confirm the pattern presented in Figure 3. Overall, self-contained methods have higher power than competitive methods, and because they test a hypothesis about single gene set, they are not affected by the proportion of gene sets in the data set that have truly DE genes (*β* parameter). Gene-level GSA approaches have slightly higher power than the other self-contained methods, followed closely by ROAST, *N*-statistic and SAM-GS, while KS has the lowest power among all self-contained methods. SeqGSEA was designed specifically for count data and it shows higher power than all other competitive methods under all settings. ROMER has relatively low power at *γ* = 0.125 but its power increases drastically at higher *γ* values, indicating it relies significantly on the proportion of DE genes in a gene set. Competitive methods are generally affected by adding more genes to the data set where adding non-DE genes enhances their power [37]. Conversely, adding DE genes may decrease it. This explains why higher *β* yields slightly lower power for ROMER especially when *γ* = 0.5 (Figure 3).

Surprisingly, unsupervised methods have low power under all settings (almost no power for GSVA). The unexpected behavior of the unsupervised methods can be explained by the sample-wise ranking they perform to calculate the enrichment scores for gene sets. To illustrate our point, consider two hypothetical cases of expression patterns in a gene set. In the first case, all DE genes in a gene set are up-regulated in phenotype 1 compared with phenotype 2. These genes group closely at the top of the ranking table for the samples under phenotype 1. The same genes have dispersed ranks for the samples under phenotype 2. This case yields high gene set enrichment score for the samples under phenotype 1 but not under phenotype 2; hence, high power is expected. Consider the second case where DE genes in a gene set are equally divided into up-regulated genes between phenotype 1 and phenotype 2. While the up-regulated genes in phenotype 1 group closely at the top of the ranking table for the samples under phenotype 1, the up-regulated genes in phenotype 2 group closely at the top of the ranking table for the samples under phenotype 2. This case yields high (however, lower than the first case) gene set enrichment score for the samples under both phenotypes; hence, low power is expected. To confirm the intuitive explanation, we implemented simulation study for two aforementioned cases. The power of both GSVA and ssGSEA is much higher when all the DE genes are up-regulated in one phenotype (see Supplementary File 2: Figure S4). Because in real data we rarely see only up-regulated or only down-regulated genes (gene sets), the power of supervised competitive methods is expected to be consistently low for real expression data. It should be noted that the authors of the ssGSEA method expected their enrichment score to be slightly more robust and more sensitive to differences in the tails of the distributions compared with the KS like statistic [41]. Our simulation results confirm this expectation.

### Robustness to samples sizes and heterogeneity (the Nigerian data set)

**Figure 4.**
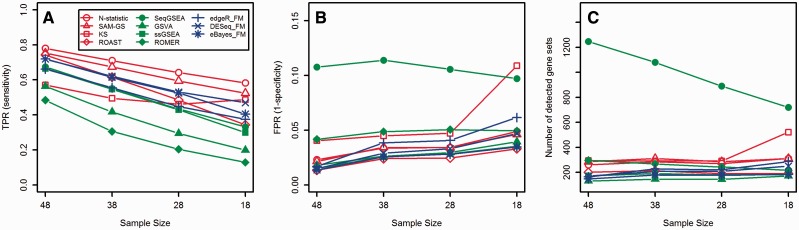
The estimated TPR (**A**), FPR (**B**) and the number of detected gene sets (**C**) by different GSA approaches. For each sample size, the results are averaged over 100 subsets composed of subsamples from the full Nigerian data set. A colour version of this figure is available at BIB online: http://bib.oxfordjournals.org.

Figure 4 shows the estimated TPR, FPR and the number of detected gene sets by different GSA approaches when 100 subsets, composed of subsamples from the full Nigerian data set, are considered (see the Methods Section for details). As expected, TPR decreases as the sample size decreases (Figure 4A). Multivariate self-contained tests (except KS) always have the highest TPR (Figure 4A), closely followed by the self-contained gene-level tests. Among competitive tests, TPR of ssGSEA and SeqGSA is less dependent on the sample size as compared with GSVA and ROMER. As the number of samples decreases, FPR slowly increases (Figure 4B), and the number of detected gene sets remains almost unchanged (Figure 4C, except KS and SeqGSEA).

**Figure 5.**
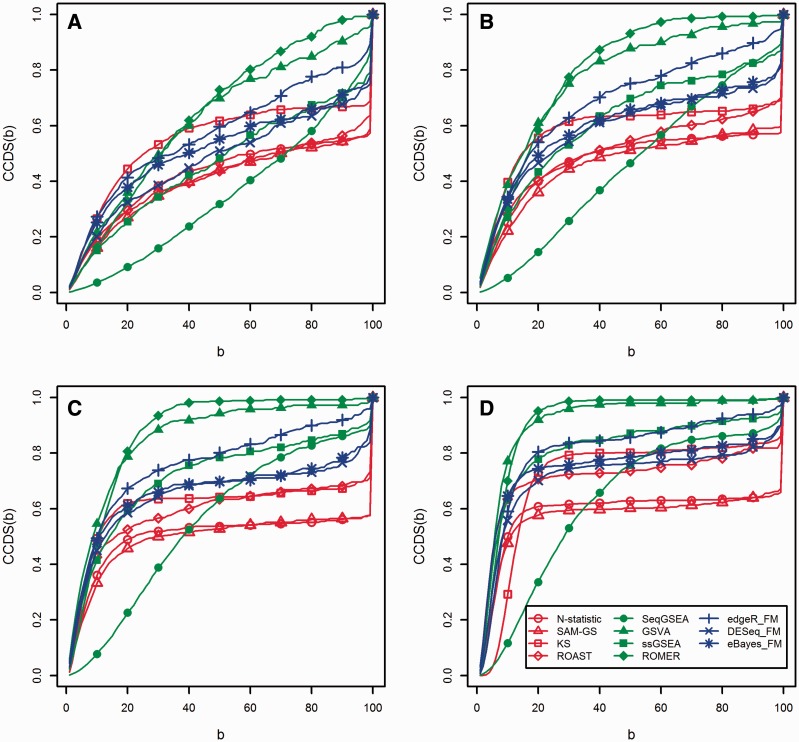
CCDS curves for different GSA approaches when 100 subsets composed of subsamples from the full Nigerian data set (58 samples) are considered with different sample sizes. (**A**) Sample size = 48; (**B**) sample size = 38; (**C**) sample size = 28; (**D**) sample size = 18. A colour version of this figure is available at BIB online: http://bib.oxfordjournals.org.

Figure 5 shows the CCDS(*b*) curves for different GSA approaches when 100 subsets, composed of subsamples from the full Nigerian data set, are considered. Again, with the exclusion of KS, multivariate self-contained tests show the highest robustness to samples heterogeneity, followed by the self-contained gene-level tests and the competitive tests. For example, at the sample size 48 (Figure 5A), about 50% of the gene sets detected by N-statistic, SAM-GS or ROAST in all 100 subsets were found in about 75% of all subsets. This proportion was reduced to 25% and 10% for egdeR_FM and ROMER, respectively. SeqGSEA persistently detects the highest number of gene sets (Figure 4C), with random overlaps between subsets, resulting in linear-like CCDF(*b*) curve for large sample sizes (48 and 38). CCDF(*b*) for SeqGSEA moves closer to the curves of other competitive methods for small sample sizes (28 and 18). GSVA and ROMER demonstrate the lowest robustness overall. Robustness of all methods decreases as the sample size decreases at a different rate, with N-statistic and SAM-GS being the most robust tests.

### The analysis of the Nigerian data set

We used the C2 gene sets to quantitatively characterize different GSA approaches based on: (i) a number of detected gene sets; (ii) the average number of genes in detected gene sets (gene set size); (iii) the proportion of DE genes in detected gene sets; and (iv) the average gene length in detected gene sets. These measures aim to highlight approaches that (i) detect too many gene sets that are not detected by the majority of other methods; (ii) detect gene sets with fewer or more genes compared with other methods (have gene set size bias); (iii) detect gene sets with larger or smaller proportion of DE genes compared with other methods (more or less sensitive); (iv) detect gene sets with higher or lower average gene length compared with other methods (have gene length bias).

Generally, self-contained methods show the highest overlap among detected DE gene sets. N-statistic, SAM-GS, KS and ROAST detect 227, 274, 260 and 202 gene sets at a significance level *α* = 0.05, where 105 gene sets are detected by all approaches (Supplementary File 2: Figure S5B). edgeR_FM, DESeq_FM and eBayes_FM detect 153, 160 and 135 gene sets, with an overlap of 94 gene sets (Supplementary File 2: Figure S5A). On the other hand, SeqGSEA, GSVA, ssGSEA and ROMER detect 1447, 113, 174 and 304 gene sets, with only 18 gene sets detected by all competitive approaches (Supplementary File 2: Figure S5C). Not surprisingly, only four gene sets are simultaneously detected by self-contained (N-statistic, ROAST and eBayes_FM) and competitive (ROMER and GSVA) approaches (Supplementary File 2: Figure S5D).

**Figure 6.**
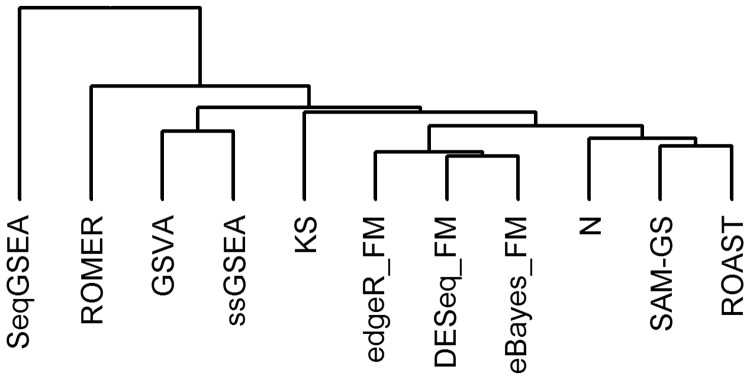
A dendrogram showing the similarity between different GSA approaches in terms of detected C2 gene sets at a significance level of 0.05. A colour version of this figure is available at BIB online: http://bib.oxfordjournals.org.

Figure 6 presents a dendrogram showing the similarity between different GSA approaches in terms of detected C2 gene sets. Self-contained and competitive tests are well separated because self-contained methods detect more gene sets in common than competitive methods. Figure 6 clearly demonstrates that the performance of various GSA methods depends on the statistical hypothesis they test, regardless of whether a method was developed for microarrays or RNA-seq data.

**Figure 7.**
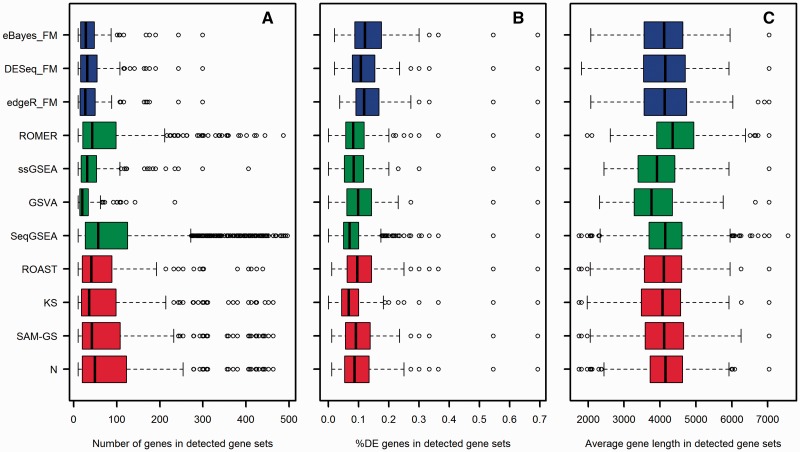
Boxplots comparing (**A**) the number of genes in gene sets (gene set size), (**B**) the proportion of DE genes in gene sets and (**C**) the average gene length per gene set in detected C2 gene sets (among 3890 C2 gene sets, *α* = 0.05) found by different GSA approaches.

Figure 7 shows boxplots for the number of genes, the proportion of DE genes and the average gene length in significantly DE C2 gene sets (among 3890 C2 gene sets, *α* = 0.05) found by different GSA approaches. The DE genes in each gene sets were detected with the empirical Bayes test (‘limma’ package [18]). The significance of the pair-wise differences between different GSA approaches (Figure 7) was evaluated using Wilcoxon’s test (Supplementary File 1: Tables S2–S4). Wilcoxon’s test (at a significance level *α* < 0.05) shows the following trends (Figure 7): (i) GSVA, ssGSEA and gene-level self-contained GSA tests detect gene sets with smaller average size as compared with all other methods; KS detects gene sets with smaller average size, as compared with other multivariate self-contained methods, SeqGSEA and ROMER; and SeqGSEA detects gene sets with larger average size, as compared with KS, GSVA, ssGSEA and gene-level self-contained methods; (ii) gene-level self-contained GSA methods detect gene sets with higher average proportion of DE genes, as compared with other methods; KS and SeqGSEA detect gene sets with lower average proportion of DE genes, as compared with other methods; and ssGSEA detects gene sets with lower average proportion of DE genes, as compared with SAM-GS, ROAST and, marginally, to KS and GSVA; (3) ROMER detects gene sets with significantly higher average gene length, as compared with all other methods, and both GSVA and ssGSEA detect gene sets with lower average gene length, as compared with other methods (respectively with low and marginal significance). These observations are summarized in Table 2.

**Table 2.** Summary of significant biases among methods

Parameter	Higher than others	Lower than others
Number of detected gene sets	SeqGSEA	None
Average gene set size	1. SegGSEA (compared with KS, GSVA, ssGSEA and gene-level self-contained methods).	1. KS, GSVA and ssGSEA.
		2. Gene-level self-contained methods.
Average %DE genes	1. Gene-level self-contained methods.	1. KS and SeqGSEA.
		2. ssGSEA (compared with SAM-GS and ROAST and marginally with KS and GSVA).
Average gene length	1. ROMER	1. GSVA (moderate significance) and ssGSEA (marginal significance).

## Discussion

A variety of GSA approaches for the analysis of microarray data has been developed. In this review, we evaluated the performance of several statistically different GSA tests (Figure 1) that can be used on RNA-seq counts data. We compared the following approaches: two nonparametric (N-statistic and KS) and one parametric (ROAST) multivariate self-contained tests; self-contained gene-level methods that (i) use RNA-seq-specific univariate tests (edgeR and DESeq) or microarray-specific test (eBayes) to combine gene-specific *P*-values into a single gene set *P*-value using FM or (ii) combine gene-specific moderated *t*-statistics in a gene set statistic using *L*_2_-norm (SAM-GS), two unsupervised competitive methods (GSVA and ssGSEA) and two supervised competitive methods (SeqGSEA and ROMER). All approaches were evaluated on simulated and real data (the Nigerian data set), and the significance levels for nonparametric tests were estimated from sample permutations (rotations for ROAST and ROMER).

We found that for simulated and real data N-statistic, SAM-GS and ROAST (multivariate self-contained methods, with the exclusion of KS) have Type I error rates near nominal *α*, have high power, are the most robust to the small sample sizes and samples heterogeneity (with minor decrease in performance for ROAST when the sample size is the smallest), have no biases in the lists of detected gene sets and detect many gene sets in common. Because ROAST and SAM-GS use moderated *t*-statistics to analyze log-scale CPM normalized counts, while N-statistic uses a different statistic to analyze log-scale RPKM normalized counts, ROAST and SAM-GS detect more gene sets in common than N-statistic (Figure 6). The KS test is the only multivariate graph-based method that ranks samples according to the structure of the MST rather than using the differences between samples in *R^p^* directly. Although such approach offers the benefit of testing a specific alternative hypothesis [24], sometimes smaller differences in *R^p^* result in larger changes in the structure of the MST and hence the ranks of the samples, as they depend on the tree traversal. This effect is further aggravated when the sample size is small and there are fewer vertices to form a tree. It makes KS more sensitive to the small sample sizes and samples heterogeneity, as compared with other multivariate methods (Figure 5). Figure 4c illustrates the sensitivity of KS to the sample size clearly: the number of KS-detected gene sets abruptly increases when 18 samples (nine males and nine females) are used. Consequently, FPR abruptly increases at the same point (Figure 4B). These observations are in agreement with the results obtained from simulated data in Table 1.

Among various approaches for combining *P*-values, FM combines *P*-values using the logarithm scale. In this way, extremely small *P*-values contribute more in a gene set statistic than large *P*-values do. Tests with FM would call a gene set DE if and only if most of the genes in a gene set have small *P*-values [25]. Gene sets with a large number of genes (large size) will be called DE less frequently than smaller gene sets because, by pure chance alone, smaller gene sets have higher probability of containing higher proportions of DE genes with extremely small *P*-values than larger gene sets (Supplementary File 2: Figure S6). This effect is further exacerbated by the fact that many gene sets in real data sets are composed of moderately or highly correlated genes that can be DE. These properties of FM coupled with biases in real data explain why gene-level GSA methods tend to detect gene sets with smaller number of genes and larger proportions of DE genes, compared with other methods in real data.

Overall, our comparative performance analyses have shown that competitive GSA approaches have less power, are highly sensitive to small sample sizes and samples heterogeneity, have tiny overlap in detected gene sets and are prone to various biases in the detected gene sets as compared with the self-contained methods. These differences stem from the difference in the hypothesis tested and the tests implementation. In particular, the gene ranking step in all competitive methods contributes to the sensitivity to samples heterogeneity and biases in detected gene sets, and in the case of unsupervised methods, allows the balance of up- and down-regulated genes in gene sets affect their power. This step also contributes to the positive correlation of Type I error rate and the number of non-DE genes [37] and the negative correlation of power and the number of DE genes (this study) in the data. While in general it is well known that the power of competitive tests is lower than the power of self-contained tests [19, 33, 39], there is no study presenting simulation scenario adequately addressing various biological parameters influencing the power of competitive and self-contained tests in the same settings.

Unsupervised competitive methods detect gene sets in the Nigerian data set with (i) fewer genes (Figure 7A) and (ii) smaller average gene length (Figure 7C) than other methods. The first bias can be explained by the sensitivity of these methods to the balance between up- and down-regulated genes in tested gene sets (Supplementary File 2: Figure S4). Small gene sets have higher probability of large imbalance between up- and down-regulated genes than large gene sets. Supplementary Figure S7 demonstrates this fact using C2 gene sets. The second bias can be explained by the specific normalization method used with unsupervised tests where distinct gene expression profiles are brought to a common scale using a discrete Poisson kernel (for RNA-seq data) without taking the differences in gene lengths into consideration. The counts of each gene are proportional to both gene abundance (molar concentration) and gene length as longer genes are expected to produce more reads in the sequencing process. Ignoring the differences in gene lengths allows shortest genes higher weights relative to the longest ones in the following sample-wise ranking step and causes the gene sets with short genes to be enriched slightly higher, and hence detected more.

ROMER analyzes VOOM-normalized data (log-scale CPM) and detects gene sets associated with any contrast in a linear model. The VOOM procedure calculates associated precision (inverse of variance) weights, which are used to account for the fact that log fold changes from genes with large counts have lower variance on the logarithm scale [41]. While package ‘limma’ incorporates these weights into its empirical Bayes pipeline within ROAST, it does not offer the same option for ROMER. ROMER ranks genes based on a moderated *t*-statistic, which normalizes the log fold change by the estimated variance plus some small positive constant. Ignoring the mean-variance trend by ROMER leads to inflated estimates of the moderated *t*-statistic for genes with larger counts relative to genes with lower counts. Because longer genes produce more reads in the sequencing process and hence larger counts, their corresponding moderated *t*-statistics are inflated and rank high relative to shorter genes. Consequently, gene sets with longer genes have high summarized enrichment scores (mean ranks) and are found by ROMER to be DE more frequently. This explains why ROMER detects gene sets with longer average gene length compared with all other methods (Figure 7C and Supplementary File 1: Table S4).

SeqGSEA detects surprisingly large numbers of gene sets in the Nigerian data set and is the most different from all the other GSA approaches (Figure 6). In addition, SeqGSEA has the highest FPR among all other methods (Figure 4B). The large number of detected gene sets by SeqGSEA affects the CCDS curve in Figure 5, which shows a linear-like pattern for sample sizes 48 and 38, and becomes slightly closer to the curves of other methods for sample sizes 28 and 18, when much less gene sets are detected (Figure 4C). These patterns collectively suggest that SeqGSEA is overlay liberal; however, such behavior was not observed with simulated data. We hypothesize that the distinct behavior of SeqGSEA can be attributed to the absence of proper normalization for the data before the ranking step. Other competitive tests do normalize the data before the analysis: both GSVA and ssGSEA use an expression-level statistic to bring expression profiles to a common scale, while ROMER uses the log-scale CPM normalization for RNA-seq counts. Both approaches shrink the dynamic range of possible gene expressions and allow smaller differences between values. Conversely, SeqGSEA uses the NB distribution to model the counts at the gene level and ranks genes based on the mean square differences between the estimated concentrations in two phenotypes normalized by the sum of their variances [38] (see Supplementary File 1 for more details). Because genes vary severely in their abundance levels (up to a few orders of magnitude), ranking them based on this statistic is unfair and makes the enrichment score highly sensitive to the few highly expressed genes in a set. The excessive sensitivity of SeqGSEA can be observed in Figure 7B where a significantly smaller proportion of DE genes is required to detect a gene set by SeqGSEA compared with other methods. In addition, the estimated variances used in the denominator of the ranking statistic are inversely proportional to the number of samples under each phenotype. When the sample size is large, the estimated variances are relatively small and the ranking statistic is generally larger for all genes, yielding larger differences and more significant enrichment scores. This argument is supported by the observed pattern in Table 1 where Type I error rate increases with the sample size. The increase in the number of detected C2 gene sets when more samples are included (Figure 4C) also supports this argument. Type I error rates in Table 1 and the power in Figure 3 were estimated based on simulated NB counts with parameters obtained from the Nigerian data set, and identical parameters were used for both phenotypes (see Methods). The simulated gene counts are smoothed versions of their real counterparts and lack over-dispersion and heterogeneity usually found in real data sets. This explains why the estimates of Type I error rate for SeqGSEA were not as large as the real data set produced. However, simulated data served its purpose of providing the flexibility to assess each method under different settings.

## Conclusions

In the ongoing transition from microarrays to RNA-seq (it appears that RNA-seq will reach the current number of arrays in GEO in 2021 [62]), it is important to know that GSA approaches developed for microarrays are equally applicable to RNA-seq data if proper normalization has been performed. The major difference between various GSA approaches, developed for microarrays or RNA-seq data remains in the null hypothesis they test and is unaffected by the data type being used. This observation is not new (e.g. [16, 22]); however, for the first time it is now also demonstrated with RNA-seq data. An important conclusion from our work that was overlooked in all former studies is that competitive GSA approaches are not robust to the samples’ heterogeneity. This means that the reproducibility of the gene sets found using competitive GSA approaches is expected to be low. This fact also contributes to the tiny overlap between gene sets found simultaneously by competitive and self-contained GSA tests. We found that, in general, the power of competitive methods depends on the number of DE genes in the data, and the power of unsupervised competitive methods in particular is influenced by the balance of up- and down-regulated genes in tested gene sets, making these methods sensitive to different gene set biases. To summarize, the best performing GSA approaches in terms of the control of the Type I error rate, power, robustness to the samples size and heterogeneity are self-contained multivariate tests such as N-statistic, ROAST and the univariate SAM-GS test that combines moderated *t*-tests in a single gene set test statistic using *L_2_* norm. These tests are easily adapted for RNA-seq data using RPKM (N-statistic) and VOOM (ROAST, SAM-GS) normalizations.

Key Points

- In this article, we compare the performance of the few GSA approaches that can be adapted from microarrays practice to fit RNA-seq data as well as those specifically designed for RNA-seq. We consider GSA approaches that cover intrinsically statistically different (in terms of null hypotheses) tests.
- GSA approaches developed for microarrays are equally applicable to RNA-seq data if proper normalization has been performed.
- Self-contained GSA tests (N-statistic, ROAST and SAM-GS) perform better than competitive supervised and unsupervised approaches (ROMER, Seq-GSEA, GSVA, ssGSEA).
- Competitive supervised and unsupervised approaches have gene set specific biases, less power and are more sensitive to the samples heterogeneity than self-contained methods, facts that have been overlooked before.

## Supplementary Data

Supplementary files are available online at http://bib.oxfordjournals.org/.

## Funding

Support has been provided in part by the National Center for Advancing Translational Science award UL1TR000039 and the IDeA Networks of Biomedical Research Excellence (INBRE) program, with grants from the National Center for Research Resources (P20RR016460) and the National Institute of General Medical Sciences (P20 GM103429) from the National Institute of Health (NIH). Large-scale computer simulations were implemented using the High Performance Computing (HPC) resources at the UALR Computational Research Center supported by the following grants: National Science Foundation grants CRI CNS-0855248, EPS-0701890, MRI CNS-0619069 and OISE-0729792. F.E.S. thanks the Engineering and Physical Research Council (EPSRC EP/H048871/1) for support.

## Supplementary Material

Supplementary Data
