# Supplementary material for: Gene set analysis approaches for RNA-seq data: performance evaluation and application guideline
Source: Brief Bioinform. 2015 Sep 4;17(3):393–407. doi: 10.1093/bib/bbv069 (PMC4870397; doi:10.1093/bib/bbv069)
Supplement: Supplementary Data [file supp_17_3_393__index.html]

Gene set analysis approaches for RNA-seq data: performance evaluation and application guideline — Gene set analysis approaches for RNA-seq data: performance evaluation and application guideline — Supplementary Data 

# Gene set analysis approaches for RNA-seq data: performance evaluation and application guideline

## Supplementary Data

files

- Supplementary Data - zip file
