# Supplementary material for: Gene set analysis approaches for RNA-seq data: performance evaluation and application guideline
Source: Brief Bioinform. 2015 Sep 4;17(3):393–407. doi: 10.1093/bib/bbv069 (PMC4870397; doi:10.1093/bib/bbv069)
Supplement: Supplementary Data [file supp_bbv069_suppl_data.zip › Supplementary file 1.pdf]

(Supplementary File 1)

Gene set analysis approaches for RNA-seq data: performance evaluation and application guideline

Yasir Rahmatallah[1], Frank Emmert-Streib[2], Galina Glazko[1,*]
[1]Division of Biomedical Informatics, University of Arkansas for Medical Sciences, Little Rock, AR 72205.
[2]Computational Medicine and Statistical Learning Laboratory, Tampere University of Technology, Korkeakoulunkatu 1, Tampere, Finland FI-33720.

ABSTRACT

This document provides supplementary materials regarding the methods, real dataset, simulation setup used in the main manuscript as well as supplementary Tables. The used statistical methods are selected representatives from different categories of gene set analysis (GSA) methods that are available in the literature. While some methods were adapted from the microarrays methodology, others were developed for the analysis of RNA-seq data. Details of preprocessing, filtering and count summarization steps of the Nigerian dataset in addition to obtaining the C2 curated gene sets from the molecular signature database are presented. The simulation setup designed to generate random Negative Binomial counts to test the effect of changing different parameters on the performance of different methods is presented.

1. Univariate Tests for RNA-seq data

There are popular parametric univariate tests used to detect differentially expressed (DE) genes between two phenotypes in RNA-seq data. These tests take into account the discrete nature of the counts that quantify gene expression abundances. We adopted three common tests: edgeR [1], DESeq [2] and eBayes [3]. In the context of GSA, the results obtained by these univariate tests for the individual genes in a gene set can be combined to obtain a gene set statistic [4] where gene-level *P*-values or test statistics can be used. The gene-level *P*-values obtained by edgeR, DESeq and eBayes were combined into gene set *P*-values in [5] using three different approaches: Fisher's combining probabilities Method (FM) [6], Stouffer's Method (SM) [7] and the soft threshold Gamma Method (GM) [8]. The performance of these three combining methods with the three univariate tests was thoroughly examined in [5]. We used FM method in this study to demonstrate the approach of combining individual genes' *P*-values. We chose FM among the three combining methods because based on the results in [5], it falls in the middle in terms of detection power, Type I error rate and biases in detected gene sets compared to SM and GM. In the following we introduce the univariate tests used in this study.

edgeR

This statistical method uses the Negative Binomial (NB) distribution to model the count dispersion of individual genes. Accurate estimation of dispersions for RNA-seq count data is critical for assessing the significance of changes in the mean expression between experimental conditions. The test employs a conditional weighted likelihood estimator to moderate individual dispersions towards a common value [1]. The amount of shrinkage is based on an approximate empirical Bayes rule. The test is suitable for small counts and small number of replicates, a situation often encountered in real experimental studies. This statistical method is implemented in the Bioconductor *edgeR* package [9].

DESeq

This statistical method also relies on the NB distribution to model the count data. It uses a generalized linear model to estimate raw variance and uses the estimated dispersion value for individual genes to fit a curve to model the mean-variance

* Correspondence: gvglazko@uams.edu

trend and allows the choice between assigning per-gene estimates and the fitted values [2]. The variance of the observed counts for gene *i* in sample *j* is modeled as

$$\sigma_{ij}^2 = \mu_{ij} + s_j^2 v_{i,\rho(j)}$$

where μ_{ij} is the expected value of the observed counts for gene i in sample j, s_j is the library size factor, $v_{i,\rho(j)}$ is the raw variance and $\rho(j)$ is the experimental condition for sample j. To normalize against differences in library sizes, this method calculates the ratios of counts in individual samples to that of a pseudo reference sample and uses the median of these ratios as the library size factor for each sample. The pseudo reference sample is the geometric mean of counts across all samples. Such procedure mitigates the strong influence a few highly and differentially expressed genes may have on the total read counts, causing it to be a biased normalization factor for the differences in library size. This statistical method is implemented in the Bioconductor *DESeq* package [2].

eBayes

This statistical method fits linear models for individual genes in the dataset and employs an empirical Bayes shrinkage procedure to moderate the standard errors of the estimated log-fold changes towards a common value. Moderating the standard errors has the effect of borrowing information among genes to aid with each gene’s inference. To find differentially expressed genes, a moderated *t*-statistic is used. This statistic has higher degrees of freedom compared to the ordinary *t*-statistic, reflecting the greater reliability associated with the smoothed standard errors [3]. eBayes was developed for microarray data and can also be used for RNA-seq data with proper normalization. This test is implemented in the Bioconductor *limma* package [10].

2. Normalization methods

Raw counts obtained in RNA-seq experiments are neither directly comparable between genes within one sample, nor between samples for the same gene [11]. This problem stems from the fact that sequencing technologies introduce technical biases which yield between-sample differences in the form of the total number of reads per sample [12] as well as within-sample variations related to the differences in gene lengths [13] and, to a lesser degree, the percent of C or G nucleotides in a gene or GC-content [14]. Raw counts are proportional to the total number of mapped reads per sample (known as library size or sequencing depth). If library sizes vary significantly between samples, the ones with larger library size are generally expected to yield larger gene counts compared to the ones with smaller library size. In addition, the counts of each gene are expected to be proportional to the gene abundance (molar concentration) and the gene length as longer genes are expected to produce more mappable reads in the sequencing process. To allow direct comparisons between gene sets in the context of differential GSA, proper count normalizations must be used to produce relative measures [15]. While univariate statistical methods require normalization against the differences between samples, some multivariate methods require normalization against both differences between samples and genes. The performance of some common normalization methods with different multivariate methods was thoroughly examined in [5] and the results demonstrated that the Type I error rate and the power of the used multivariate tests depended mainly on the test statistics and are insensitive to the different normalizations. Some statistical methods (univariate or multivariate) recommend and implement specific normalization methods.

It is worth mentioning here that as a result of the process of alternative splicing the final processed messenger RNA produced from a gene may include or lack particular exons of that gene. Therefore, different isoforms (with different lengths) of the same gene may be expressed in sequenced samples. It was estimated that about 95% of multiexonic genes are alternatively spliced in humans [16]. Therefore, care must be taken when gene length information is employed for normalizing raw counts. One approach for obtaining RNA-seq expression levels in the form of discrete counts is to summarize mapped reads directly for genes regardless of different isoforms, i.e. when the genomic coordinates of a read overlaps any of the exons of a unique gene this read is counted towards that gene (e.g. the HTSeq pipeline [17]). In this case there are many options to extract gene length information such as using the length of the longest isoform, the median isoform length or the sum of the lengths of exons in the gene. An alternative approach for obtaining RNA-seq counts is to summarize counts for isoforms and then combine the counts of the isoforms belonging to the same gene to obtain its total expression abundance (e.g. the Cufflinks pipeline [18]). In this case normalization takes place in the isoform level before summing the counts of the isoforms belonging to the same gene. This approach however yields normalized expressions rather than discrete counts which limits the use of statistical methods that require raw counts and use their own recommended normalizations. We adopted the first approach and used the length of the longest isoform per gene as gene length as provided by the Bioconductor *GSVAdata* package [19]. This data package provides annotation data for human genes defined by Entrez identifiers and downloaded from the National Center for Biotechnology Information (NCBI) on 22/02/2012. In the following we introduce some of the normalization methods employed in this study.

Reads per kilo base per million (RPKM)

The reads per kilo base per million (RPKM) mapped reads measure normalizes raw gene counts by the total number of mapped reads in each sample (library size) and by the gene lengths [12]. It is popular, simple and one of the first proposed

normalization procedures for count data. In one sample, the number of mapped reads to gene g, n_g, with length L_g base pairs can be normalized using the RPKM measure as

$$RPKM_g = \frac{n_g \times 10^9}{L_g \times N}$$

where N is the total number of mapped reads in the sample. This normalization facilitates the comparison of abundance levels both between and within samples.

Trimmed mean of M-values (TMM)

The trimmed mean of M-values (TMM) is an empirical normalization strategy that estimates sample normalization factors which attempts to equates the overall expression levels of genes between samples under the assumption that the majority of the genes in the dataset are not differentially expressed [20]. It uses a weighted trimmed mean of the log expression ratio between the samples and one selected pseudo reference sample. To provide a robust estimation, the upper and lower values of the log expression ratio and absolute expression levels are trimmed before calculating the weighted average. After trimming, a weighted mean of the log expression ratio is calculated, with the inverse of the approximate asymptotic variances used as weights to account for the fact that genes with larger counts have log expression ratios with lower variance on the logarithm scale [20]. According to the hypothesis that the majority of the genes in the dataset are not differentially expressed, TMM should be close to 1. If the hypothesis is false (TMM≠1), TMM values provide an estimate of the normalization factors that must be applied to the raw counts in each sample to fulfil the hypothesis, hence it accounts for the differences in library size. The TMM normalization is implemented in the Bioconductor *edgeR* package [9].

Variance modelling at the observational level (VOOM)

This normalization method was originally motivated by the desire of applying normal-based microarray-like statistical methods to RNA-seq count data based on the idea that it is more important to model the mean-variance trend of RNA-seq counts correctly than it is to determine the exact distribution of the counts [21]. It opens access to use a large body of methodology originally developed for microarrays. This method starts by fitting gene-level linear models to the log-scale counts per million (CPM) of the raw counts normalized for library sizes. Then, a robust trend fits the generated residual standard deviations by the linear models as a function of the average log-CPM. The mean-variance trend is incorporated to predict the variance of each log-CPM observation. The inverse predicted variance for each observation is incorporated as an associated weight into the linear model to account for the mean-variance trend [21]. This feature makes VOOM especially suited for the *limma* empirical eBayes pipeline [3], the rotation gene set test (ROAST) [22] and the rotation testing using mean ranks (ROMER) [23]. This normalization method as well as the three mentioned methods (eBayes, ROAST and ROMER) that can make best use of it are all implemented in the Bioconductor *limma* package [10].

It is worth mentioning that transformations such as VOOM which convert the negative binomial count data into quasi-normal normalized data are suitable with GSA methods that aggregate gene-level statistics or *P*-values to form a statistic for each gene set. They are not suitable for GSA methods where the expressions of different genes are directly compared against each other. The reason behind is that such transformations (like VOOM) do not account for the differences in gene length allowing longer genes higher leverage over shorter genes (gene count is proportional to gene length). Hence we do not recommend count transformations (such as VOOM) for methods that compare gene expressions directly (such as N-statistic) or rank the expressions of different genes under the same sample (such as GSVA or ssGSEA).

3. Gene set analysis methods

The use of gene set analysis (GSA) methods was motivated by three benefits: First, small and moderate changes in gene expressions of many genes cannot be captured by conventional univariate methods that test individual genes separately [24]; Second, genes interact with each other and hence accounting for the multivariate nature of expression changes is more biologically relevant [25, 26]; Third, gene sets may represent molecular pathways, biological processes or candidate genes which gives them explanatory power as differential gene sets offer better biological explanation compared to individual genes. GSA approaches are classified into two categories: *competitive* and *self-contained* [27, 28]. Competitive approaches test the hypothesis that gene expression levels in a gene set are associated with phonotypes as much as the expression levels of the dataset outside that gene set, while self-contained approaches test the hypothesis that gene expression levels in a gene set are not associated with the phenotype. Both approaches have their own benefits and pitfalls. Self-contained approaches have been reported to have higher detection power than competitive approaches, but they have the potential weakness of testing inequivalent hypotheses [26]. Some competitive approaches are influenced by the genomic coverage and the filtering steps applied to the dataset and have been reported to increase their power by the addition of unrelated data and noise [29].

Although not truly multivariate, the univariate statistical methods introduced earlier (edgeR, DESeq and eBayes), followed by aggregating individual genes' *P*-values to obtain gene set *P*-values fall into the category of self-contained approaches. Besides combining *P*-values, some methods combine the gene-level statistics to obtain gene set statistics and estimate significance through sample permutations. In what follows we summarize the self-contained and competitive methods used in this study. Statistically speaking the problem of detecting differentially expressed gene sets in a dataset is a hypothe-

sis testing problem that can be formulated as having two different phenotypes with n_1 samples of measurements of p genes for the first and n_2 samples of measurement of the same p genes for the second, $N=n_1+n_2$. The samples under the two phenotypes consist of p-dimensional random vectors of measurements $\boldsymbol{X}=(X_1,\ldots, X_{n1})$ and $\boldsymbol{Y}=(Y_1,\ldots, Y_{n2})$ that are independent and identically distributed with the distribution functions F_x, F_y, mean vectors μ_x, μ_y and $p\times p$ covariance matrices S_x , S_y. Depending on the test statistic, we can test the general hypothesis H_0: $F_x = F_y$ against an alternative H_1: $F_x \neq F_y$, or a restricted hypothesis H_0: $\mu_x = \mu_y$ against an alternative H_1: $\mu_x \neq \mu_y$.

3.1 Self-contained multivariate tests

We present a method that uses the differences in R^p space between the p-dimensional samples under two different phenotypes. We also present a method that uses the differences in sample ranks based on a graph with a tree structure. Finally, we present a method which uses the framework of linear models and test the hypothesis that all the model coefficients equal to zero.

N-statistic

A two-sample nonparametric multivariate test can be implemented using the N-statistic [30, 31]. The test statistic is the difference of the sum of all the distances between the p-dimensional samples from the two different phenotypes and the two corresponding sums of distances of the p-dimensional samples within the same sample (with normalization factors applied to all terms). We use the statistic:

$$N_{n_1n_2} = \frac{n_1n_2}{n_1+n_2}\left[\frac{1}{n_1n_2}\sum_{i=1}^{n_1}\sum_{j=1}^{n_2} L(X_i,Y_j) - \frac{1}{2n_1^2}\sum_{i=1}^{n_1}\sum_{j=1}^{n_2} L(X_i,X_j) - \frac{1}{2n_2^2}\sum_{i=1}^{n_1}\sum_{j=1}^{n_2} L(Y_i,Y_j)\right]^{1/2}$$

where we consider only $L(X,Y) = \|X-Y\|$, the Euclidian distance in R^p. This statistic tests the general hypothesis that H_0: $F_x = F_y$ against a two-sided alternative H_1: $F_x \neq F_y$. The asymptotic null distribution of the test statistic is derived by permuting sample labels and calculating the statistic for a large number of times. We used 1000 permutations to achieve acceptable estimate. Raw counts were normalized with RPKM and normalized expressions Y_{ij} are transformed to log-scale using the transformation $\log_2(1+Y_{ij})$ before applying this test. The implementation of this test in R is available within the CRAN *cramer* package (http://cran.r-project.org/web/packages/cramer/index.html).

Multivariate Kolmogorov-Smirnov (KS)

We adopted the multivariate generalization of the Kolmogorov-Smirnov (KS) test [32] as suggested by Friedman and Rafsky [33]. The performance of this method in the context of GSA with RNA-seq data was examined in [5]. The multivariate generalization is based on the minimum spanning tree (MST) of the complete undirectional edge-weighted graph generated from the gene expression data. Normalized expressions were obtained using the RPKM normalization to normalize against differences in library size and gene length and normalized expressions Y_{ij} are transformed to log-scale using the transformation $\log_2(1+Y_{ij})$ before applying this test. The test starts by forming an undirectional edge-weighted graph $G(V,E)$ where V is the set of vertices (p-dimensional observations) and E is the set of edges. The MST is defined as the acyclic subset of edges $T\subseteq E$ that connects all vertices in V with minimal total length. For the p-dimensional observations X and Y, the edge-weighted complete graph can be constructed, with N vertices and $N(N$-1)/2 edges with weights estimated by the Euclidean (or any other) distance measure between pairs of points in R^p. The MST of such graph connects all N vertices that are close in R^p with N-1 edges.

The vertices are ranked based on their MST to obtain the strong relation between vertices differences in ranks and their distances in R^p. The MST is rooted at a node with the largest geodesic distance (one of the two nodes connected by the longest path in the MST) and then the nodes are ranked in the high directed preorder (HDP) traversal of the tree starting from the root [33]. The quantity $d_i = r_i/n_1 - s_i/n_2$ is calculated where r_i (s_i) is the number of observations in X (Y) ranked lower than i, $1 \leq i \leq N$. The test statistic is the maximal absolute difference $D = \max_i|d_i|$ and the null hypothesis is rejected for large D [33]. This method tests the restricted hypothesis H_0: $\mu_x = \mu_y$ against an alternative H_1: $\mu_x \neq \mu_y$. The null distribution of the test statistic D is estimated using sample permutation strategy where the phenotype labels of the vertices (samples) in the MST are randomly permuted for M=1000 times and D is calculated. P-value is defined as the proportion of permutations that produce more extreme statistics than the observed one and is calculated as

$$p-value = \frac{\sum_{i=1}^{M} I[D_i > D_{observed}] + 1}{(M+1)}$$

The implementation of the multivariate generalization of the Kolmogorov-Smirnov test in R was released within the Bioconductor *GSAR* package.

Rotation gene set test (ROAST)

The rotation gene set test (ROAST) is a parametric multivariate statistical test that was originally proposed for GSA of microarrays [22]. ROAST uses the framework of linear models and tests if the expression levels of the genes in a set yield a

particular non-zero contrast of the model coefficients [22]. It accounts for correlations between genes and can use different alternative hypotheses, testing whether the direction of changes for genes in a set is *up*, *down* or *mixed* (up or down) [22]. For all comparisons implemented here the *mixed* hypothesis was selected. Instead of permutation, ROAST assess the significance using rotation, a Monte Carlo simulation scheme for multivariate regression models [34]. Since the number of rotations does not depend on sample size, ROAST gives useful results and avoids the problem of granularity of P-values even for experiments with small sample size. ROAST constructs a gene set statistic from the summary of the moderated t-statistics for the genes in the set. Among a few possible summary statistics, we used the mean of the squared gene-level statistics. This choice is more sensitive than the others when only a few genes in the set are DE, or if some genes have much larger log fold changes than others.

Using ROAST with RNA-seq count data requires proper normalization. The VOOM normalization [21] was proposed specifically for this purpose where log counts per millions, normalized for library size are used. In addition to counts normalization, VOOM calculates associated precision weights which can be incorporated into the linear modeling process within ROAST to eliminate the mean-variance trend in the normalized counts [21].

3.2 Self-contained methods that combine gene-level statistics

We present a method that aggregates gene-level statistics to obtain gene set statistics and estimates significance in a nonparametric manner through sample label permutations.

Significance analysis of microarrays-gene sets (SAM-GS)

This two-sample nonparametric method was originally suggested for microarrays by Dinu *et al* [35] to extend the use of the individual-gene method Significance Analysis of Microarrays (SAM) [36] to gene sets. We extend this method to RNA-seq count data by applying the VOOM normalization [21] to find the log-scale CPM of the raw counts normalized for library sizes prior to the test. The test statistic is the L_2-norm of the moderated t-statistic for the gene expressions

$$T_{SAM-GS} = \sum_{i=1}^{p} \left(\frac{\bar{X}_i - \bar{Y}_i}{s_i + s_0} \right)^2$$

where $\bar{X}_i$ and $\bar{Y}_i$ are respectively the mean expression levels for gene i under phenotypes X and Y, s_i is a pooled standard deviation over the samples in the two phenotype, s_0 is a small positive constant to adjust for small variability and p is the number of genes in the gene set. This statistic tests the restricted hypothesis H_0: $\mu_x = \mu_y$ against an alternative H_1: $\mu_x \neq \mu_y$. The null distribution of the test statistic is estimated by permuting sample labels and calculating the statistic for 1000 times. The significance (P-value) is calculated as the proportion of permutations that yield more extreme statistics than the observed statistic. The SAM-GS method was implemented in R by the authors of the method and the code is available online at (https://www.ualberta.ca/~yyasui/software.html#SAM-GS).

3.3 Self-contained methods that combine gene-level P-values

Most of the available differential analysis methods for RNA-seq count data produce significance measures (P-values) by testing hypotheses for individual genes. In the context of GSA, gene-level P-values can be aggregated in order to provide a combined P-value for the gene set. This combined value can be used to test the overall hypothesis applied to the gene set [4]. This approach agrees with the general aims of GSA to group genes in sets to facilitate biological interpretation, increase detection power and detect a gene set when multiple genes have small or moderate changes between different phenotypes. Generally, P-value combining methods transform the P-values obtained by testing L hypotheses at the gene-level by some function H and then aggregate the transformed values (possibly using weights w_is) to calculate the combined value, $T = \sum_{i=1}^{p} w_i H(P_i)$, where p is the number of genes in the gene set. If weights are not used, all w_is are set to $1/p$.

Three common combining methods in the literature are Fisher's combined probability method [6], Stouffer's inverse normal method [7] and soft truncation threshold Gamma method [8]. These methods transform gene-level P-values respectively using the natural logarithm, the inverse standard normal cumulative distribution function and the inverse gamma cumulative distribution function. These different transforms emphasize small (significant) P-values in variable degrees relative to large (insignificant) P-values. The higher the emphasis to small P-values, the smaller the proportion of DE genes required to deem a gene set differentially expressed. This normally leads to higher detection power and Type I error rate. The performance and sensitivity of these three combining methods to different parameters and biases was thoroughly examined in [5]. We chose Fisher' method to demonstrate the approach of combining individual gene P-values among the three combining methods because based on the results in [5], it falls in the middle in terms of power, Type I error rate and biases in detected gene sets. Fisher's method transforms P-values using the natural logarithm and the combined value is given by

$$T = -2 \sum_{i=1}^{p} w_i \, log_e(P_i)$$

When all p null hypotheses are true and independent, T follows a chi-square distribution with $2p$ degrees of freedom [6]. When p is very large, this test statistic favors P-values smaller than $1/e \approx 0.368$ such that

$$P_c \to \begin{cases} 0 & P_i < 1/e\,, \forall i \\ 1 & P_i > 1/e\,, \forall i \\ 1/e & P_i = 1/e\,, \forall i \end{cases}$$

where P_c is the combined P-value of the gene set. However, this threshold point is a little smaller than $1/e$ for a finite value of p. The combined P-value P_c is computed using the distribution of the combined value T. However, due to the lack of independence between genes in a gene set, parametric approaches are rendered inaccurate. The lack of independence among genes stems from the fact that genes do not work in isolation and often show different levels of correlation. Instead, we estimate the null distribution of T using a nonparametric approach based on random permutations of sample labels to preserve the correlation structure between genes. We estimate the gene-level P-values and T for 1000 random permutations to get a reasonable estimate of the null distribution. The empirical estimate of the combined P-value P_c is the proportion of the permutations in which T was found to be larger than the observed value.

3.4 Competitive supervised methods

These approaches test the hypothesis that gene expression levels in a gene set are associated with phonotypes as much as the expression levels of the dataset outside that gene set. The phenotype labels are used during the calculation of the enrichment score (ES) for gene sets. We present one method that was developed for the analysis of RNA-seq data and another than was developed for microarrays and adapted to RNA-seq data here after normalizing the counts.

SeqGSEA

The first competitive statistical method suggested for testing differential expression for a set of genes in microarray experiments was the Gene Set Enrichment Analysis (GSEA) [24, 37]. Since its inception, many variations of GSEA were proposed in the literature. The SeqGSEA method was proposed with the aim of integrating the differential expression (DE) and differential splicing (DS) analyses from RNA-seq count data with functional GSA [38]. This method was implemented in the Bioconductor *SeqGSEA* package [39]. This approach uses the NB distribution to model the counts at the gene level (similar to edgeR or DESeq). The raw count for gene i and sample j, Y_{ij}, can be represented as $Y_{ij} \sim NB(\mu_{ij}, \sigma_{ij}^2)$, where $\mu_{ij} = s_j q_{i,\rho(j)}$. The mean parameter μ_{ij} is the product of the size factor s_j indicating the library size for sample j and $q_{i,\rho(j)}$ is the expected concentration of reads from gene i under condition $\rho(j)$. The DE score for gene i between groups X and Y is defined as

$$SCORE_{DE}(i) = \frac{\left(\hat{q}_{i,X} - \hat{q}_{i,Y}\right)^2}{\hat{V}\left(q_{i,X}\right) + \hat{V}\left(q_{i,Y}\right)}$$

where $\hat{q}_{i,X}$ and $\hat{q}_{i,Y}$ are respectively the estimate of the expected expression $q_{i,\rho(j)}$ for gene i under groups X and Y, and $\hat{V}\left(q_{i,X}\right)$ and $\hat{V}\left(q_{i,Y}\right)$ respectively denotes the variance estimate of $q_{i,\rho(j)}$ under groups X and Y. The significance of the test is found in a nonparametric manner using sample permutations. The DE score is also normalized by the mean of the scores obtained through permutations.

The method calculates enrichment scores for both differential expression and splicing and combine these two scores using two possible strategies (linear combination or rank-based strategy) to obtain integrated gene set enrichment analysis [38]. However, since we are interested in DE analysis only, we followed the exemplified pipeline for such analysis as suggested in the package vignette [39]. According to [38], the gene expression levels obtained from RNA-seq counts are generally not well variance stabilized which yield degenerate results when the proposed score is used for DE analysis only. This can be mainly attributed to the amplification of the instability in gene-level statistic by the ranking process. Therefore, we expect that the gene sets found to be DE by SeqGSEA to be less reliable than the ones found by other methods which better accommodate the nature of the data. This expectation was confirmed later using a real dataset where SeqGSEA produced unreliable results (see Results and Discussion sections in the main manuscript).

Rotation testing using mean ranks (ROMER)

The rotation testing using mean ranks (ROMER) is a parametric multivariate competitive GSEA method that uses the framework of linear models [10, 23]. It finds gene sets associated with a non-zero contrast of the coefficients in the linear model. Similar to ROAST [22], it estimates the significance based on rotations [34] instead of permutations and therefore also works well with a small number of samples. It also accounts for correlations between genes and tests whether the direction of change is *up*, *down* or *mixed*. Since the direction of change in gene sets is unknown, we tested the mixed hypothesis only. ROMER constructs a gene set statistic by summarizing the moderated t-statistics for the genes in the set using the mean function. Before applying ROMER to RNA-seq data, counts were normalized with VOOM [21] to obtain log CPM expressions values normalized for library size. ROMER is implemented in the Bioconductor *limma* package [10]. Both ROAST and ROMER were developed originally for microarray data. ROAST was adapted for RNA-seq counts through the use of VOOM normalization and made use of the estimated weights by VOOM and incorporated them into the linear modeling process to

account for the mean-variance trend in the normalized counts. In contrast to ROAST, the *limma* implementation of ROMER does not incorporate these weights.

3.5 Competitive unsupervised methods

These methods summarize the enrichment activity score for gene sets under each sample regardless of phenotype labels (hence the term unsupervised). Then, standard analytical methods can be applied to detect differential enrichment. We present two methods that differ only in the summary statistic they use to calculate the enrichment scores. While one uses a Kolmogorov-Smirnov random walk approach, the other uses the weighted average ranks.

Gene set variation analysis (GSVA)

Gene Set Variation Analysis (GSVA) is a nonparametric, unsupervised method for performing gene set enrichment analysis in microarray and RNA-seq gene expression data. Unlike most GSEA methods, it transforms the data from a gene by sample matrix to a gene set by sample matrix, thereby allowing the evaluation of gene set enrichment for each sample and the application of standard analytical methods for gene set scores [40]. The method starts by bringing individual gene expression profiles to a common scale by calculating an expression level statistic through nonparametric kernel estimation of the cumulative density functions of these profiles. While a Gaussian kernel [41] is used for microarray continuous data, a discrete Poisson kernel [42] is employed for RNA-seq count data. Second, the statistic is converted to ranks for each sample and scaled to have the ranks centered around zero. Finally, an enrichment score is calculated using the Kolmogorov-Smirnov like random walk statistic. Two approaches are available to calculate the ES: First, a maximum deviation from the zero score similar to the classical GSEA [37], yielding an ES with bimodal distribution. Second, the difference between the largest positive and negative random walk deviations from zero, yielding a ES with a standard normal-like distribution. We used the later ES in this work. This method is implemented in the Bioconductor *GSVA* package [40]. The empirical Bayes method [3] from package *limma* [10] was used to find the DE gene sets from the resulted gene set by sample matrix (gene set enrichment matrix).

Single-sample gene set enrichment analysis (ssGSEA)

This is a nonparametric method which uses exactly the same procedure as GSVA and produces a gene set by sample enrichment matrix. It differs from GSVA only by the enrichment score (ES) it employs. The ES for a gene set under one sample is obtained by the sum of the difference between the weighted empirical cumulative distribution functions of the genes inside the set and outside the set [43]. In other words, it employs the weighted average ranks instead of the maximum deviation Kolmogorov–Smirnov like random walk statistic used in GAVA. This ES was claimed to be slightly more robust and more sensitive to differences in the tails of the distributions compared to the Kolmogorov–Smirnov like statistic. This makes it more suited to represent the gene set enrichment for sets with relatively few genes attaining high expression values [43]. The Bioconductor *GSVA* package offers an implementation for this method [40].

4. Real dataset and gene sets

This section presents the real dataset and simulated count data used to assess the performance of the GSA methods. Details of the preprocessing steps implemented for the real dataset and the simulation setup are provided. We also refer to the Bioconductor package used to perform each step.

Preprocessing the Nigerian dataset

In this study, we demonstrated the behavior of different statistical methods using a subset of the Pickrell [14] dataset of sequenced cDNA libraries generated from 69 lymphoblastoid cell lines that were derived from Yoruban Nigerian individuals (YRI) as part of the HapMap project. We selected this dataset due to its adequate sample size and its balanced phenotypes (has almost the same number of male and female samples).

Raw sequenced RNA-seq short reads in FASTQ file format are available at [44]. We picked only the samples sequenced at the Yale sequencing center using the Illumina Genome Analyzer 2 platform. Among available samples, we considered only 58 unrelated individual (parents) with 29 males and 29 females. Raw reads (of length 35 base pairs) were aligned to the UCSC hg19 human genome using the Bowtie aligner [45] without allowing mismatches. Supplementary Table S1 summarizes the numbers and percentages of the reads successfully aligned by Bowtie and mapped uniquely to an annotated gene. The lists of exonic coordinates grouped by genes from the hg19 human genome model were obtained using the Bioconductor *GenomicFeatures* package (version 1.18.3) [46]. All genes were given Entrez identifiers. The Bioconductor *GenomicAlignments* package (version 1.2.1) [44] with “Union” counting mode was used to summarize mapped reads by genes, where reads overlapping one of the exons that belong uniquely to one gene are counted towards that gene. Reads overlapping more than one gene were discarded. The obtained count matrix had counts mapped to a total of 26258 annotated genes with the library sizes ranging between 0.894×10^6 and 3.64×10^6.

The obtained count matrix is further filtered to ensure only expressed genes are considered. We filter out any gene which does not satisfy any of two conditions: (*i*) have nonzero counts in more than 20 samples out of the total 58; (*ii*) have a mean counts per million (CPM) greater than 1. We used the length of the longest isoform as gene length information as provided by the Bioconductor *GSVAdata* package (version 1.1.1) [19]. This data package provides annotation data for human genes de-

fined by Entrez identifiers and downloaded from NCBI on 22/02/2012. Any genes with no supported gene length information were discarded. Finally, Entrez identifiers were replaced by unique Symbol identifiers using the Bioconductor *org.Hs.eg.db* annotation package (version 3.0.0) [47]. The resulted count matrix has a total of 13251 annotated genes and 58 samples (29 males and 29 females) with library sizes ranging between 0.881×10^6 and 3.613×10^6.

C2 curated gene sets from the molecular signatures database

Meaningful groups of genes are necessary to conduct any GSA. We used the C2 group of gene sets from the molecular signature database (MSigDB) 4.0 [37]. These gene sets (a total of 4722) were collected from various sources such as online pathway databases, publications in PubMed, and knowledge of experts. The list of gene sets was downloaded and accessed in R using the Bioconductor *GSEABase* package (version 1.28.0) [48]. We considered only the genes present in both the filtered Nigerian count matrix and the list of C2 gene sets. We also considered only C2 sets with p genes where $10\leq p\leq500$. After these filtering steps, the count matrix was left with 11903 genes in 58 samples and 3890 C2 gene sets to analyze.

5. Simulations setup

Generating random counts

We model the count for gene i in sample j by a random variable Y_{ij} with Negative Binomial (NB) distribution

$$Y_{ij}\sim NB\big(mean=\mu_{ij}, var=\mu_{ij}(1+\mu_{ij}\varphi_{ij})\big)=NB\big(\mu_{ij},\varphi_{ij}\big)$$

where μ_{ij} and φ_{ij} are respectively the mean count and dispersion parameter of gene i in sample j. For each gene in a gene set, a vector of mean count, dispersion and gene length information (μ_i,φ_i,L_i) is randomly picked from a pool of vectors derived from the processed Nigerian dataset. To provide uniform set of samples and avoid possible extreme samples while estimating the mean and dispersion parameters, we considered genes which have nonzero counts in more than 20 samples out of the total 58, have counts per millions between 1 and 1000 and used a subset of 44 samples for which the library size ranged between 1.85×10^6 and 3.15×10^6. Dispersion parameters for individual genes were estimated using the Bioconductor *edgeR* package (version 3.8.5) [9] as follows: First, library size of the 44 samples was reduced to that of the sample with minimum library size by binomial thinning. Second, dispersion parameters for individual genes were estimated by an empirical Bayes method based on weighted conditional maximum likelihood [1]. The obtained pool of parameters from the reduced Nigerian dataset has 13251 vectors (μ_i,φ_i,L_i). Negative Binomial random counts can be generated from the obtained pool of vectors and later normalized with any method. The generation of synthetic counts for simulation purposes does not require an accurate estimation of μ_i and φ_i for each gene but simply to provide a plausible distribution of values to represent the typical abundances and variation due to biological and technical effects [49].

Estimating Type I error rates from simulated data

Random counts following the $NB(\mu_i,\varphi_i)$ distribution were generated using the pool of parameter vectors obtained earlier from the reduced Nigerian dataset (consisting of 44 samples). To simulate the null hypothesis H_0: $F_x=F_y$, we generated a dataset consisting of N samples (equally separated into two phenotypes) and 1000 gene sets of equal size (p). Hence, we have a dataset of N samples and $1000\times p$ genes. The randomly selected parameter vector (μ_i,φ_i,L_i) is used to generate NB counts for gene i under all the samples in the dataset. To examine the effects of sample size and gene set size, we estimated Type I error rate under different parameter settings for all statistical methods. We chose $p\in\{16, 60, 100\}$ and $N\in\{10, 20, 40, 60\}$. Type I error rate for a statistical test is calculated as the proportion of gene sets detected by the test when the null hypothesis H_0: $F_x=F_y$ is true. The results were averaged over ten generated datasets to obtain more stable results.

Estimating power from simulated data

In real data, differentially expressed gene set may include genes that are up-regulated, down-regulated, similarly expressed between two phenotypes, with variable fold change. Therefore, to mimic real data as closely as possible three simulation parameters were introduced: β, the proportion of gene sets in the dataset that have truly differentially expressed genes; γ, the percentage of genes, truly differentially expressed in each gene set and FC, the fold change in gene counts between two phenotypes. We consider $\beta\in\{0.05, 0.25\}$ and $\gamma\in\{0.125, 0.25, 0.5\}$. For the parameter FC, the values are in the range [1.2, 3]. To represent two biological conditions with different outcomes two groups with equal sample size, $N/2$ (N=20 and N=40) were considered. For each group S=1000 non-overlapping gene sets, each constructed from p random realizations of NB distribution, were formed. Relatively small (p=16) and large (p=100) gene set size were chosen. The power for all methods was estimated by testing the hypothesis H_0: $\mu_x=\mu_y$ (or H_0: $FC=1$) against an alternative H_1: $\mu_x\neq\mu_y$ (or H_1: $FC\neq1$) for all gene sets. For each of the $(1-\beta)S$ non-DE gene sets p random realizations of $NB(\mu_i,\varphi_i)$ were sampled, where $1\leq i\leq p$ under both phenotypes. For each of the βS gene sets that have truly DE genes, $p/2$ random realizations of $NB(\mu_i,\varphi_i)$ and $NB(FC\,\mu_i,\varphi_i)$ were sampled, under phenotype 1 and phenotype 2 for $1\leq i\leq\gamma p/2$. Also $p/2$ random realizations of $NB(FC\,\mu_i,\varphi_i)$ and $NB(\mu_i,\varphi_i)$ were sampled under phenotype 1 and phenotype 2 for $(\gamma p/2)+1\leq i\leq\gamma p$. In this way half of the γp DE genes in each gene set were up-regulated and half were down-regulated between the two phenotypes. In real data, the situation where almost all the DE genes in a gene set are up-regulated under one phenotype is extremely rare and rather there are some genes

that are up-regulated under one phenotype and others that are up-regulated under another. Therefore, the way we simulate gene counts with distributing the fold change effect over both phenotypes mimic real data closer than the case of applying the fold change effect under one phenotype.

REFERENCES

1. Robinson MD, Smyth GK, **Moderated statistical tests for assessing differences in tag abundance.** *Bioinformatics* 2007, **23:**2881-2887.
2. Anders S, Huber W, **Differential expression analysis for sequence count data.** *Genome biology* 2010, **11:**R106.
3. Smyth GK, **Linear models and empirical Bayes methods for assessing differential expression in microarray experiments.** *Stat Appl Genet Mol Biol* 2004, **3**:3.
4. Ackermann M, Strimmer K, **A general modular framework for gene set enrichment analysis.** *BMC Bioinformatics* 2009, **10**(1):47.
5. Rahmatallah Y, Emmert-Streib F, Glazko G, **Comparative evaluation of gene set analysis approaches for RNA-Seq data.** *BMC Bioinformatics* 2014, **15**:397.
6. Fisher R, *Statistical methods for research workers.* Edinburgh, Scotland, Oliver and Boyd; 1932.
7. Stouffer S, DeVinney L, Suchmen E, *The American Soldier: Adjustment during army life.* Princeton, US: Princeton University Press; 1949.
8. Fridley BL, Jenkins GD, Grill DE, Kennedy RB, Poland GA, Oberg AL, **Soft truncation thresholding for gene set analysis of RNA-seq data: application to a vaccine study.** *Sci Rep* 2013, **3:**2898.
9. Robinson MD, McCarthy DJ, Smyth GK, **edgeR: a Bioconductor package for differential expression analysis of digital gene expression data.** *Bioinformatics* 2010, **26:**139-140.
10. Ritchie ME, Phipson B, Wu D, Hu Y, Law CW, Shi W, Smyth GK, **limma powers differential expression analyses for RNA-sequencing and microarray studies.** *Nucleic Acid Research* 2015, **1**.
11. Risso D, Schwartz K, Sherlock G, Dudoit S, **GC-Content Normalization for RNA-Seq Data.** *BMC Bioinformatics* 2011, **12**:480.
12. Mortazavi A, Williams BA, McCue K, Schaeffer L, Wold B, **Mapping and quantifying mammalian transcriptomes by RNA-Seq.** *Nature methods* 2008, **5:**621-628.
13. Oshlack A, Robinson MD, Young MD, **From RNA-seq reads to differential expression results.** *Genome biology* 2010, **11:**220.
14. Pickrell JK, Marioni JC, Pai AA, Degner JF, Engelhardt BE, Nkadori E, Veyrieras JB, Stephens M, Gilad Y, Pritchard JK, **Understanding mechanisms underlying human gene expression variation with RNA sequencing.** *Nature* 2010, **464:**768-772.
15. Dillies MA, Rau A, Aubert J, Hennequet-Antier C, Jeanmougin M, Servant N, Keime C, Marot G, Castel D, Estelle J, Guernec G, Jagla B, Jouneau L, Laloë D, Le Gall C, Schaëffer B, Le Crom S, Guedj M, Jaffrézic F, French StatOmique Consortium, **A comprehensive evaluation of normalization methods for Illumina high-throughput RNA sequencing data analysis.** *Briefings in bioinformatics* 2012.
16. Pan Q, Shai O, Lee LJ, Frey BJ, Blencowe BJ, **Deep surveying of alternative splicing complexity in the human transcriptome by high-throughput sequencing.** *Nature Genetics* 2008, **40** (12): 1413–1415.
17. Anders S, Pyl PT, Huber W, **HTSeq—a Python framework to work with high-throughput sequencing data.** *Bioinformatics* 2015, **31**(2): 166-169.
18. Trapnell C, Roberts A, Goff L, Pertea G, Kim D, Kelley DR, Pimentel H, Salzberg SL, Rinn JL, Pachter L, **Differential gene and transcript expression analysis of RNA-seq experiments with TopHat and Cufflinks.** *Nature Protocols* 2012, **7**: 562-578
19. Castelo R, *GSVAdata: Data employed in the vignette of the GSVA package*. R package version 1.1.1.
20. Robinson MD, Oshlack A, **A scaling normalization method for differential expression analysis of RNA-seq data.** *Genome biology* 2010, **11:**R25.
21. Law CW, Chen Y, Shi W, Smyth GK, **Voom: precision weights unlock linear model analysis tools for RNA-seq read counts.** *Genome Biol* 2014, **15:**R29.
22. Wu D, Lim E, Vaillant F, Asselin-Labat ML, Visvader JE, Smyth GK, **ROAST: rotation gene set tests for complex microarray experiments.** *Bioinformatics* 2010, **26:**2176-2182.
23. Majewski IJ, Ritchie ME, Phipson B, Corbin J, Pakusch M, Ebert A, Busslinger M, Koseki H, Hu Y, Smyth GK, Alexander WS, Hilton DJ, Blewitt ME, **Opposing roles of polycomb repressive complexes in hematopoietic stem and progenitor cells.** *Blood* 2010, **116**(5): 731-739.
24. Mootha VK, Lindgren CM, Eriksson KF, Subramanian A, Sihag S, Lehar J, Puigserver P, Carlsson E, Ridderstrale M, Laurila E, Houstis N, Daly MJ, Patterson N, Mesirov JP, Golub TR, Tamayo P, Spiegelman B, Lander ES, Hirschhorn JN, Altshuler D, Groop LC, **PGC-1alpha-responsive genes involved in oxidative phosphorylation are coordinately downregulated in human diabetes**. *Nat Genet* 2003, **34**(3):267-273.
25. Glazko GV, Emmert-Streib F, **Unite and conquer: univariate and multivariate approaches for finding differentially expressed gene sets**. *Bioinformatics* 2009, **25**(18):2348-2354.
26. Emmert-Streib F, Glazko GV, **Pathway analysis of expression data: deciphering functional building blocks of complex diseases**. *PLoS Comput Biol* 2011, **7**(5):e1002053.
27. Goeman JJ, Buhlmann P, **Analyzing gene expression data in terms of gene sets: methodological issues**. *Bioinformatics* 2007, **23**(8):980-987.

28. Tian L, Greenberg SA, Kong SW, Altschuler J, Kohane IS, Park PJ, **Discovering statistically significant pathways in expression profiling studies**. *Proc Natl Acad Sci USA* 2005, **102**(38):13544-13549.
29. Tripathi S, Glazko GV, Emmert-Streib F, **Ensuring the statistical soundness of competitive gene set approaches: gene filtering and genome-scale coverage are essential**. *Nucleic Acids Res* 2013, **41**(7):e82.
30. Baringhaus L, Franz C, **On a new multivariate two-sample test**. *Journal of Multivariate Analysis* 2004, **88**:190-206.
31. Klebanov L, Glazko G, Salzman P, Yakovlev A, Xiao Y, **A multivariate extension of the gene set enrichment analysis**. *J Bioinform Comput Biol* 2007, **5**(5):1139-1153.
32. Rahmatallah Y, Emmert-Streib F, Glazko G, **Gene set analysis for self-contained tests: complex null and specific alternative hypotheses**. *Bioinformatics* 2012, **28**(23):3073-3080.
33. Friedman JH, Rafsky C, **Multivariate Generalizations of the Wald-Wolfowitz and Smirnov Two-Sample Tests.** *The Annals of Statistics* 1979, **7**(4): 697-717.
34. Langsrud Ø, **Rotation tests.** *Statistics and Computing* 2005, **15**(1): 53–60.
35. Dinu I, Potter JD, Mueller T, Liu Q, Adewale AJ, Jhangri GS, Einecke G, Famulski KS, Halloran P and Yasui Y, **Improving gene set analysis of microarray data by SAM-GS.** *BMC Bioinformatics* 2007, **8**:242.
36. Tusher VG, Tibshirani R, Chu G, **Significance analysis of microarrays applied to the ionizing radiation response.** *Proc Natl Acad Sci USA* 2001, **98**, 5116-5121.
37. Subramanian A, Tamayo P, Mootha VK, Mukherjee S, Ebert BL, Gillette MA, Paulovich A, Pomeroy SL, Golub TR, Lander ES and Mesirov JP, **Gene set enrichment analysis: a knowledge-based approach for interpreting genome-wide expression profiles.** *Proc Natl Acad Sci USA* 2005, **102**: 15545-15550.
38. Wang X and Cairns MJ, **Gene Set Enrichment Analysis of RNA-Seq Data: Integrating Differential Expression and Splicing.** *BMC Bioinformatics* 2013, **14**(Suppl 5): S16.
39. Wang X and Cairns MJ, **SeqGSEA: a Bioconductor package for gene set enrichment analysis of RNA-Seq data integrating differential expression and splicing.** *Bioinformatics* 2014, **30**(12): 1777-1779.
40. Hänzelmann S, Castelo R, Guinney J, **GSVA: gene set variation analysis for microarray and RNA-seq data.** *BMC Bioinformatics* 2013, **14**:7.
41. Silverman B, *Density Estimation for Statistics and Data Analysis*. Chapman and Hall; 1986.
42. Canale A, Dunson DB, **Bayesian kernel mixtures for counts.** *Journal of the American Statistical Association* 2011, **106**(496):1528-1539.
43. Barbie DA, Tamayo P, Boehm JS, Kim SY, Moody SE, Dunn IF, Schinzel AC, Sandy P, Meylan E, Scholl C, Fröhling S, Chan EM, Sos ML, Michel K, Mermel C, Silver SJ, Weir BA, Reiling JH, Sheng Q, Gupta PB, Wadlow RC, Le H, Hoersch S, Wittner BS, Ramaswamy S, Livingston DM, Sabatini DM, Meyerson M, Thomas RK, Lander ES, Mesirov JP, Root DE, Gilliland DG, Jacks T and Hahn WC, **Systematic RNA interference reveals that oncogenic KRAS-driven cancers require TBK1.** *Nature* 2009, **462**:108-112.
44. **Raw unmapped RNA-Seq short reads in FASTQ format files.** [http://eqtl.uchicago.edu/RNA_Seq_data/unmapped_reads/]
45. Langmead B, Trapnell C, Pop M, Salzberg SL, **Ultrafast and memory-efficient alignment of short DNA sequences to the human genome.** *Genome Biol* 2009, **10:**R25.
46. Lawrence M, Huber W, Pagès H, Aboyoun P, Carlson M, Gentleman R, Morgan M and Carey V, **Software for Computing and Annotating Genomic Ranges.** *PLoS Computational Biology* 2013, **9**:8.
47. Carlson M, *org.Hs.eg.db: Genome wide annotation for Human*. R package version 3.0.0.
48. Morgan M, Falcon S and Gentleman R, *GSEABase: Gene set enrichment data structures and methods*. R package version 1.28.0.
49. Robles JA, Qureshi SE, Stephen SJ, Wilson SR, Burden CJ, Taylor JM, **Efficient experimental design and analysis strategies for the detection of differential expression using RNA-Sequencing.** *BMC Genomics* 2012, **13:**484.

Table S1. Short reads alignment summary for samples belonging to unrelated Nigerian individuals

Sample	Nigerian Yale sample (35 bp short reads)	Gender (M/F)	# Reads processed (FASTQ)	# Reported alignments (by Bowtie)	% Reported alignments (by Bowtie)	# Alignments to a unique hg19 gene	% Alignments to a unique hg19 gene
1	NA18501_yale	M	6422000	5838083	90.91	2079066	35.61
2	NA18502_yale	F	8909231	7976026	89.53	2723006	34.14
3	NA18504_yale	M	7113128	6380433	89.70	2155282	33.78
4	NA18505_yale	F	8849477	8057799	91.05	2624343	32.57
5	NA18507_yale	M	10329429	9219847	89.26	3128985	33.94
6	NA18508_yale	F	8701858	7896491	90.74	2695741	34.14
7	NA18516_yale	M	7818182	7158331	91.56	2371240	33.13
8	NA18517_yale	F	9367811	8513394	90.88	2992649	35.15
9	NA18522_yale	M	7691810	6968610	90.60	2538127	36.42
10	NA18523_yale	F	4528349	3796290	83.83	1312807	34.58
11	NA18852_yale	F	9606138	8571651	89.23	2977933	34.74
12	NA18853_yale	M	9717230	8967320	92.28	2009494	22.41
13	NA18855_yale	F	7930075	6943915	87.56	2465620	35.51
14	NA18856_yale	M	5267997	4719944	89.60	1549829	32.84
15	NA18858_yale	F	8800059	7884153	89.59	2763487	35.05
16	NA18859_yale	M	10007505	8750612	87.44	3186117	36.41
17	NA18861_yale	F	4642527	4228902	91.09	1543821	36.51
18	NA18862_yale	M	8619671	7799474	90.48	2362129	30.29
19	NA18870_yale	F	6833330	6079199	88.96	2105333	34.63
20	NA18871_yale	M	6261611	5497420	87.80	1882764	34.25
21	NA18912_yale	F	4211405	3905879	92.75	1144997	29.31
22	NA18913_yale	M	9006101	7960151	88.39	2721949	34.19
23	NA19092_2_yale	M	11123460	9840519	88.47	3643071	37.02
24	NA19093_yale	F	6997219	6342108	90.64	2305299	36.35
25	NA19098_yale	M	10280860	9381273	91.25	3363836	35.86
26	NA19099_yale	F	9955015	8956531	89.97	3200150	35.73
27	NA19101_yale	M	9320429	8497804	91.17	2900625	34.13
28	NA19102_yale	F	4025513	3748758	93.12	1098147	29.29
29	NA19116_yale	F	9693662	8872576	91.53	2902698	32.72
30	NA19119_yale	M	3816178	3508443	91.94	894677	25.50
31	NA19127_yale	F	9353324	8563060	91.55	2462348	28.76
32	NA19128_yale	M	9498521	8850191	93.17	2620720	29.61
33	NA19130_yale	M	8914041	8200243	91.99	2798086	34.12
34	NA19131_yale	F	8414765	7669283	91.14	2657974	34.66
35	NA19137_yale	F	6574947	5798550	88.19	2173162	37.48
36	NA19138_yale	M	9041280	8050926	89.05	2909436	36.14
37	NA19140_yale	F	8970177	8098705	90.28	2649404	32.71
38	NA19141_yale	M	9626823	8654012	89.89	3156669	36.48
39	NA19143_yale	F	8381930	7619857	90.91	2535320	33.27
40	NA19144_yale	M	8327861	7575682	90.97	2768315	36.54
41	NA19152_yale	F	10247210	9327922	91.03	3234596	34.68
42	NA19153_yale	M	8474542	7714514	91.03	2706509	35.08
43	NA19159_yale	F	6382899	5684534	89.06	2143404	37.71
44	NA19160_yale	M	9662020	8700495	90.05	2835799	32.59
45	NA19171_yale	M	6154300	5529208	89.84	1949075	35.25
46	NA19172_yale	F	7086910	6395504	90.24	2156739	33.72
47	NA19192_yale	M	8552871	7637761	89.30	2598569	34.02
48	NA19193_yale	F	9370200	8470026	90.39	2283219	26.96
49	NA19200_yale	M	6213671	5642583	90.81	1944585	34.46
50	NA19201_yale	F	9408711	8611509	91.53	2927061	33.99
51	NA19203_yale	M	9630700	8685162	90.18	2572915	29.62
52	NA19204_yale	F	6353169	5627854	88.58	2066252	36.71
53	NA19206_2_yale	F	11010264	9624047	87.41	3329304	34.59
54	NA19207_yale	M	11044860	9917916	89.80	3546600	35.76
55	NA19209_yale	F	10301610	9403400	91.28	3201941	34.05
56	NA19210_yale	M	9956027	9091087	91.31	2960352	32.56
57	NA19238_yale	F	8406706	7657214	91.08	2552332	33.33
58	NA19239_yale	M	9085407	8279008	91.12	2813211	33.98

Table S2. Wicoxon's test *P*-values for the average number of genes in detected C2 gene sets

	N	SAMGS	KS	ROAST	SeqGSEA	GSVA	ssGSEA	ROMER	edgeR_FM	DESeq_FM	eBayes_FM
**N**	1.0E+00	7.9E-01	2.4E-06	5.5E-01	6.8E-01	9.6E-12	3.4E-09	2.2E-01	6.2E-03	1.7E-02	8.8E-03
**SAMGS**	7.9E-01	1.0E+00	2.1E-06	7.1E-01	9.6E-01	3.3E-12	1.8E-09	3.2E-01	7.7E-03	2.4E-02	1.1E-02
**KS**	2.4E-06	2.1E-06	1.0E+00	2.5E-05	1.9E-08	1.2E-02	1.6E-01	6.7E-04	3.4E-02	1.8E-02	4.1E-02
**ROAST**	5.5E-01	7.1E-01	2.5E-05	1.0E+00	6.5E-01	2.8E-11	3.4E-08	5.9E-01	2.1E-02	6.3E-02	2.7E-02
**SeqGSEA**	6.8E-01	9.6E-01	1.9E-08	6.5E-01	1.0E+00	1.0E-13	8.0E-12	2.4E-01	2.8E-03	9.2E-03	4.6E-03
**GSVA**	9.6E-12	3.3E-12	1.2E-02	2.8E-11	1.0E-13	1.0E+00	1.9E-01	3.4E-08	1.5E-06	4.7E-07	2.5E-06
**ssGSEA**	3.4E-09	1.8E-09	1.6E-01	3.4E-08	8.0E-12	1.9E-01	1.0E+00	2.7E-06	5.8E-04	1.9E-04	8.7E-04
**ROMER**	2.2E-01	3.2E-01	6.7E-04	5.9E-01	2.4E-01	3.4E-08	2.7E-06	1.0E+00	1.2E-01	2.2E-01	1.4E-01
**edgeR_FM**	6.2E-03	7.7E-03	3.4E-02	2.1E-02	2.8E-03	1.5E-06	5.8E-04	1.2E-01	1.0E+00	6.7E-01	9.8E-01
**DESeq_FM**	1.7E-02	2.4E-02	1.8E-02	6.3E-02	9.2E-03	4.7E-07	1.9E-04	2.2E-01	6.7E-01	1.0E+00	6.8E-01
**eBayes_FM**	8.8E-03	1.1E-02	4.1E-02	2.7E-02	4.6E-03	2.5E-06	8.7E-04	1.4E-01	9.8E-01	6.8E-01	1.0E+00

Table S3. Wicoxon's test *P*-values for the average proportion of DE genes in detected C2 gene sets

	N	SAMGS	KS	ROAST	SeqGSEA	GSVA	ssGSEA	ROMER	edgeR_FM	DESeq_FM	eBayes_FM
**N**	1.0E+00	4.6E-01	8.9E-05	1.0E-01	2.5E-05	4.8E-01	9.7E-02	4.4E-01	1.4E-07	5.4E-05	1.4E-06
**SAMGS**	4.6E-01	1.0E+00	6.6E-07	3.0E-01	4.9E-09	8.5E-01	1.4E-02	7.2E-02	8.2E-07	3.2E-04	6.4E-06
**KS**	8.9E-05	6.6E-07	1.0E+00	4.3E-08	2.6E-01	1.6E-04	4.3E-02	2.7E-04	2.6E-19	8.1E-15	9.9E-17
**ROAST**	1.0E-01	3.0E-01	4.3E-08	1.0E+00	1.9E-10	5.6E-01	1.4E-03	6.9E-03	2.6E-04	1.5E-02	8.9E-04
**SeqGSEA**	2.5E-05	4.9E-09	2.6E-01	1.9E-10	1.0E+00	4.7E-05	5.4E-02	2.6E-05	7.9E-28	8.7E-21	6.0E-23
**GSVA**	4.8E-01	8.5E-01	1.6E-04	5.6E-01	4.7E-05	1.0E+00	3.2E-02	9.7E-02	5.4E-04	1.4E-02	1.2E-03
**ssGSEA**	9.7E-02	1.4E-02	4.3E-02	1.4E-03	5.4E-02	3.2E-02	1.0E+00	3.9E-01	2.8E-11	5.0E-08	7.0E-10
**ROMER**	4.4E-01	7.2E-02	2.7E-04	6.9E-03	2.6E-05	9.7E-02	3.9E-01	1.0E+00	2.6E-12	3.3E-08	1.9E-10
**edgeR_FM**	1.4E-07	8.2E-07	2.6E-19	2.6E-04	7.9E-28	5.4E-04	2.8E-11	2.6E-12	1.0E+00	2.1E-01	9.3E-01
**DESeq_FM**	5.4E-05	3.2E-04	8.1E-15	1.5E-02	8.7E-21	1.4E-02	5.0E-08	3.3E-08	2.1E-01	1.0E+00	2.8E-01
**eBayes_FM**	1.4E-06	6.4E-06	9.9E-17	8.9E-04	6.0E-23	1.2E-03	7.0E-10	1.9E-10	9.3E-01	2.8E-01	1.0E+00

Table S4. Wicoxon's test *P*-values for the average gene length in detected C2 gene sets

	N	SAMGS	KS	ROAST	SeqGSEA	GSVA	ssGSEA	ROMER	edgeR_FM	DESeq_FM	eBayes_FM
**N**	1.0E+00	5.8E-01	1.3E-01	5.2E-01	8.9E-01	5.2E-04	1.2E-02	4.9E-04	1.0E+00	7.6E-01	5.8E-01
**SAMGS**	5.8E-01	1.0E+00	3.0E-01	8.8E-01	3.3E-01	2.0E-03	4.2E-02	2.5E-05	6.6E-01	8.8E-01	9.0E-01
**KS**	1.3E-01	3.0E-01	1.0E+00	4.5E-01	1.8E-02	4.0E-02	3.7E-01	5.2E-07	2.1E-01	3.2E-01	4.7E-01
**ROAST**	5.2E-01	8.8E-01	4.5E-01	1.0E+00	2.9E-01	6.6E-03	9.6E-02	7.6E-05	5.8E-01	7.7E-01	9.8E-01
**SeqGSEA**	8.9E-01	3.3E-01	1.8E-02	2.9E-01	1.0E+00	1.0E-05	5.4E-04	1.8E-06	8.2E-01	5.6E-01	3.8E-01
**GSVA**	5.2E-04	2.0E-03	4.0E-02	6.6E-03	1.0E-05	1.0E+00	1.5E-01	5.1E-09	5.8E-03	7.9E-03	1.0E-02
**ssGSEA**	1.2E-02	4.2E-02	3.7E-01	9.6E-02	5.4E-04	1.5E-01	1.0E+00	3.6E-08	6.3E-02	8.5E-02	1.2E-01
**ROMER**	4.9E-04	2.5E-05	5.2E-07	7.6E-05	1.8E-06	5.1E-09	3.6E-08	1.0E+00	3.5E-03	7.8E-04	4.5E-04
**edgeR_FM**	1.0E+00	6.6E-01	2.1E-01	5.8E-01	8.2E-01	5.8E-03	6.3E-02	3.5E-03	1.0E+00	8.0E-01	6.4E-01
**DESeq_FM**	7.6E-01	8.8E-01	3.2E-01	7.7E-01	5.6E-01	7.9E-03	8.5E-02	7.8E-04	8.0E-01	1.0E+00	8.2E-01
**eBayes_FM**	5.8E-01	9.0E-01	4.7E-01	9.8E-01	3.8E-01	1.0E-02	1.2E-01	4.5E-04	6.4E-01	8.2E-01	1.0E+00
