# Supplementary material for: Gene set analysis approaches for RNA-seq data: performance evaluation and application guideline
Source: Brief Bioinform. 2015 Sep 4;17(3):393–407. doi: 10.1093/bib/bbv069 (PMC4870397; doi:10.1093/bib/bbv069)
Supplement: Supplementary Data [file supp_bbv069_suppl_data.zip › Supplementary file 2.pdf]

Gene set analysis approaches for RNA-seq data: performance evaluation and application guideline (Supplementary File 2)

Yasir Rahmatallah [1], Frank Emmert-Streib [2] and Galina Glazko [1]
[1] Department of Biomedical Informatics,
University of Arkansas for Medical Sciences,
Little Rock, AR 72205.
[2] Computational Medicine and Statistical Learning Laboratory,
Tampere University of Technology,
Korkeakoulunkatu 1, Tampere, Finland FI-33720.

July 9, 2015

List of Figures

S1 The power of different tests to detect differences between two groups of samples when the alternative hypothesis (H_1) holds true with different settings (values of β, γ and FC). The gene set size is $p = 100$ and the sample size in each group is $N/2$ ($N = 20$). 2
S2 The power of different tests to detect differences between two groups of samples when the alternative hypothesis (H_1) holds true with different settings (values of β, γ and FC). The gene set size is $p = 16$ and the sample size in each group is $N/2$ ($N = 40$). . 3
S3 The power of different tests to detect differences between two groups of samples when the alternative hypothesis (H_1) holds true with different settings (values of β, γ and FC). The gene set size is $p = 100$ and the sample size in each group is $N/2$ ($N = 40$). 4
S4 The power of unsupervised competitive tests (GSVA and ssGSEA) to detect differences between two groups of samples when the alternative hypothesis (H_1) holds true with different settings (values of β, γ and FC). The gene set size is $p = 16$ and the sample size in each group is $N/2$ ($N = 20$). In case 1, all the $\gamma \times p$ DE genes in a gene set are up-regulated in phenotype 1 compared to phenotype 2. In case 2, half of the $\gamma \times p$ DE genes in a gene set are up-regulated in phenotype 1 and the other half are up-regulated in phenotype 2. Both GSVA and ssGSEA have much higher power under case 1. . . . 5
S5 Venn diagrams showing the number of common C2 gene sets detected in the processed Nigerian dataset by different GSA methods ($\alpha = 0.05$) considering a total of 3890 gene sets. (a) Gene-level self-contained methods (edgeR, DESeq and eBayes with Fisher's method for combining P-values); (b) multivariate self-contained methods (N-statistic, SAM-GS, KS and ROAST); (c) competitive methods (SeqGSEA, GSVA, ssGSEA and ROMER); (d) mixture of methods (N-statistic, ROAST, eBayes with Fisher's method, GSVA and ROMER). 6
S6 The proportion of differentially expressed (DE) genes found by eBayes in each of the 3890 C2 gene sets at a significance level $\alpha = 0.05$ versus gene set size for the Nigerian dataset. 7
S7 Difference between the number of up and down-regulated genes in each C2 gene set normalized by its size. (a) Using eBayes over VOOM-normalized data; (b) using edgeR over count data. Analyzing the Nigerian dataset in both panels show that small C2 gene sets have higher differences between the number of up and down-regulated genes than large C2 gene sets. 8

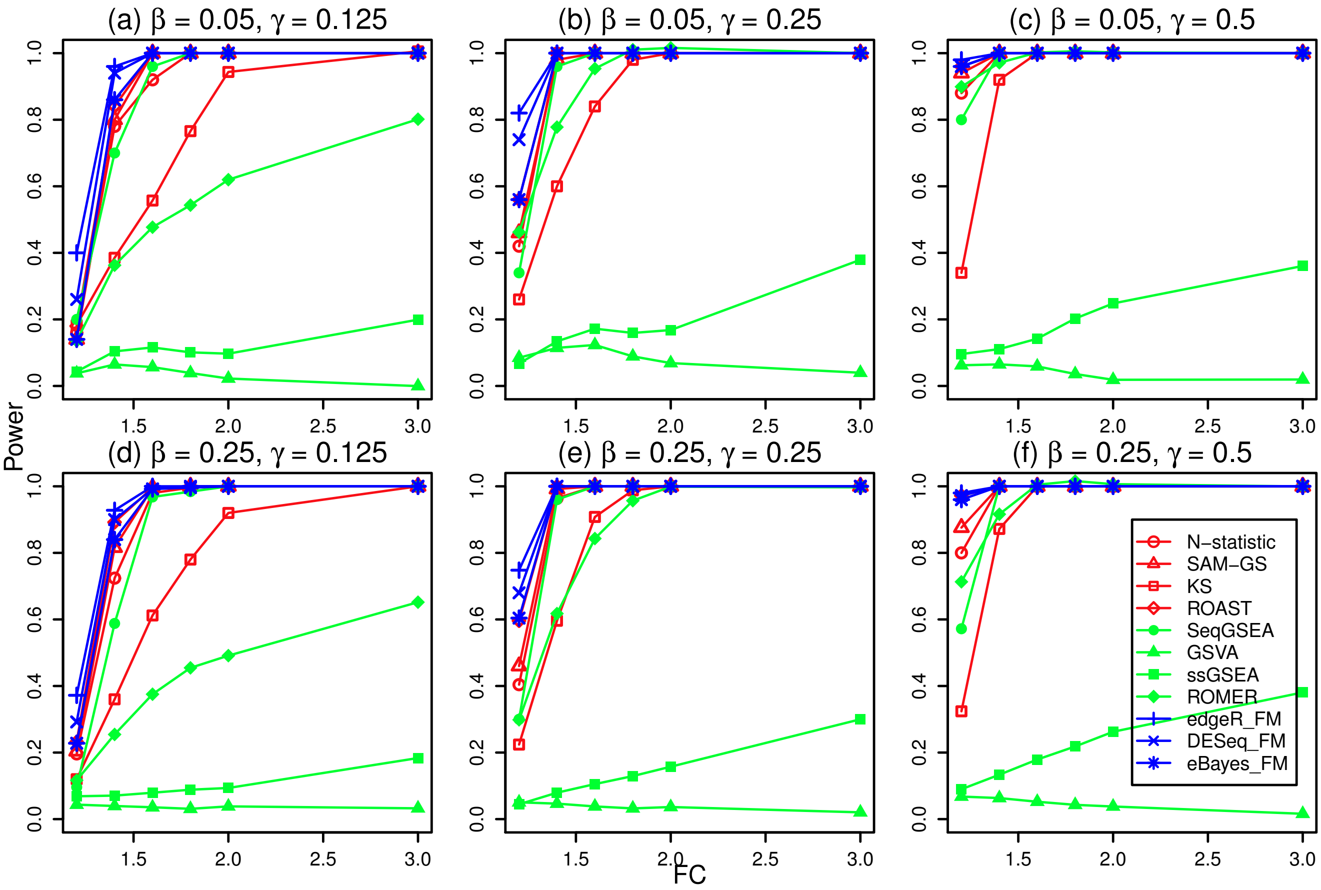

Figure S1: The power of different tests to detect differences between two groups of samples when the alternative hypothesis (H_1) holds true with different settings (values of β, γ and FC). The gene set size is $p = 100$ and the sample size in each group is $N/2$ ($N = 20$).

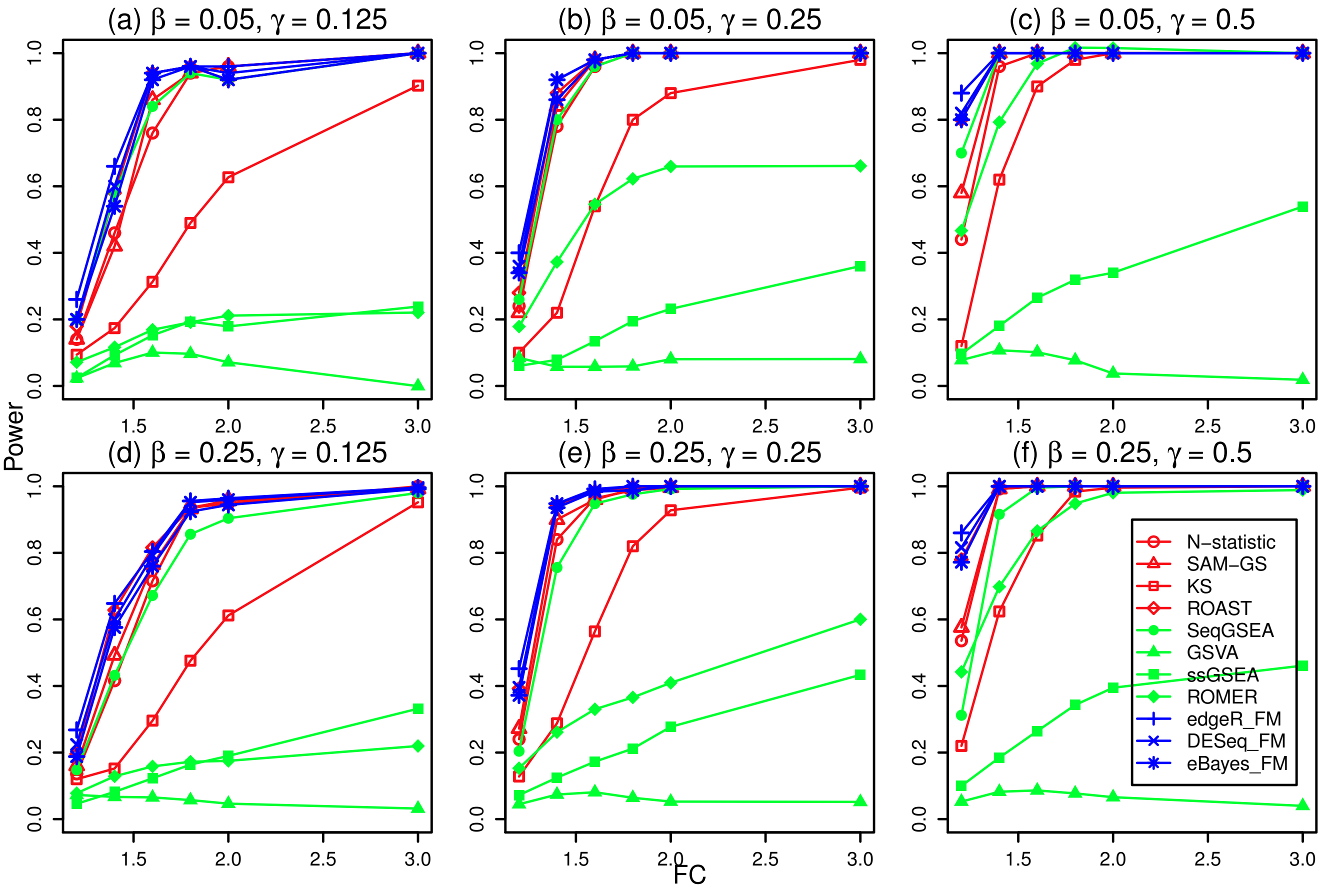

Figure S2: The power of different tests to detect differences between two groups of samples when the alternative hypothesis (H_1) holds true with different settings (values of β, γ and FC). The gene set size is $p = 16$ and the sample size in each group is $N/2$ ($N = 40$).

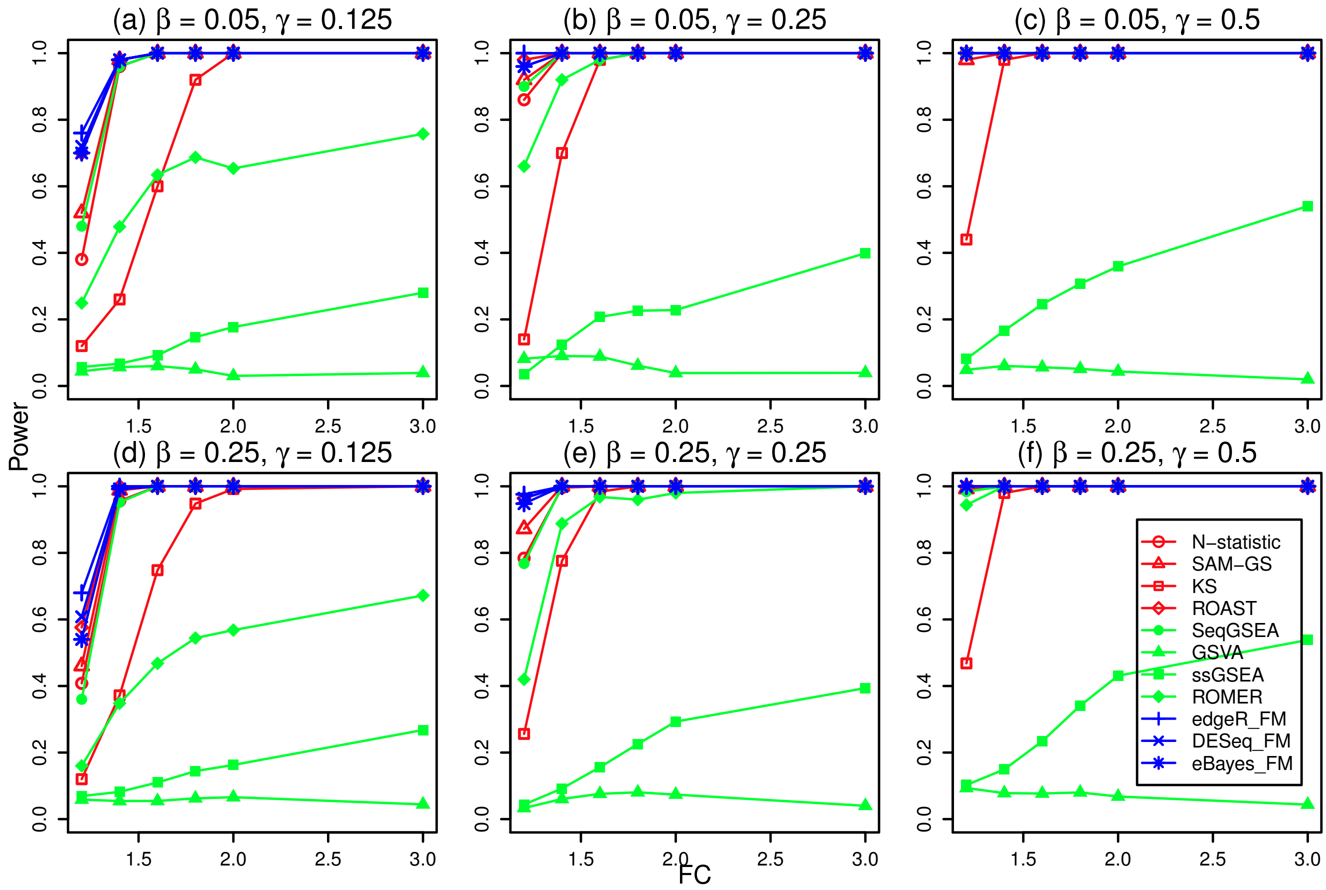

Figure S3: The power of different tests to detect differences between two groups of samples when the alternative hypothesis (H_1) holds true with different settings (values of β, γ and FC). The gene set size is $p = 100$ and the sample size in each group is $N/2$ ($N = 40$).

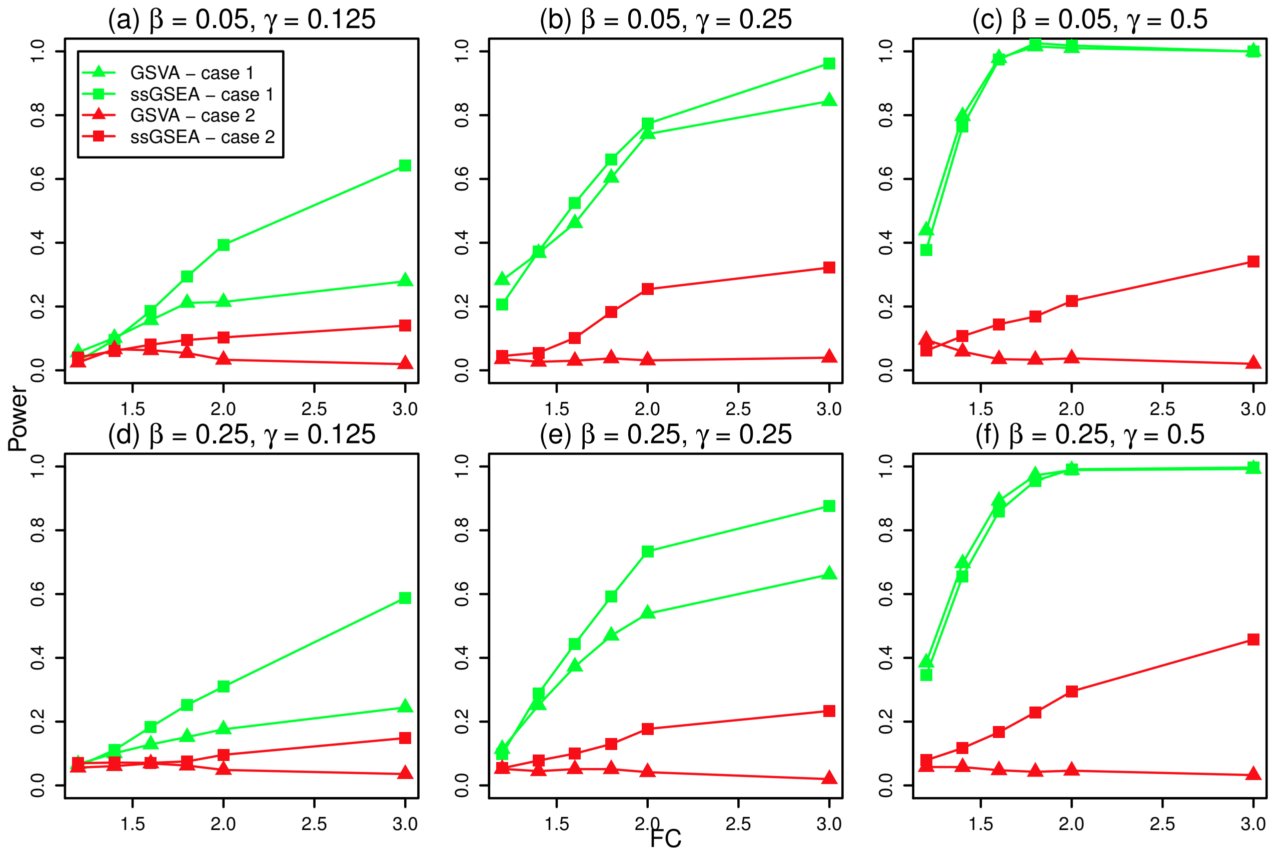

Figure S4: The power of unsupervised competitive tests (GSVA and ssGSEA) to detect differences between two groups of samples when the alternative hypothesis (H_1) holds true with different settings (values of β, γ and FC). The gene set size is $p = 16$ and the sample size in each group is $N/2$ ($N = 20$). In case 1, all the $\gamma \times p$ DE genes in a gene set are up-regulated in phenotype 1 compared to phenotype 2. In case 2, half of the $\gamma \times p$ DE genes in a gene set are up-regulated in phenotype 1 and the other half are up-regulated in phenotype 2. Both GSVA and ssGSEA have much higher power under case 1.

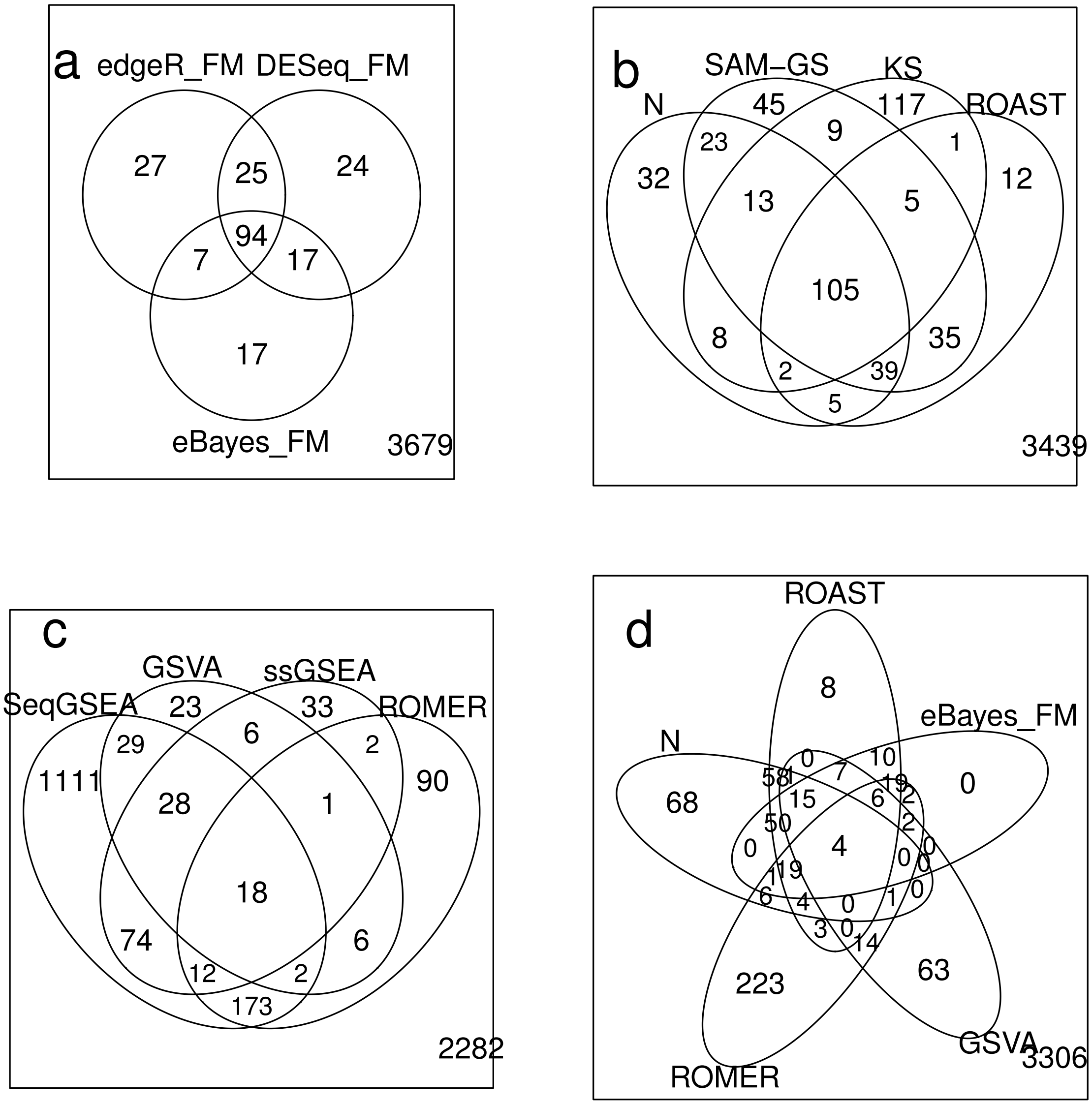

Figure S5: Venn diagrams showing the number of common C2 gene sets detected in the processed Nigerian dataset by different GSA methods ($\alpha = 0.05$) considering a total of 3890 gene sets. (a) Gene-level self-contained methods (edgeR, DESeq and eBayes with Fisher's method for combining P-values); (b) multivariate self-contained methods (N-statistic, SAM-GS, KS and ROAST); (c) competitive methods (SeqGSEA, GSVA, ssGSEA and ROMER); (d) mixture of methods (N-statistic, ROAST, eBayes with Fisher's method, GSVA and ROMER).

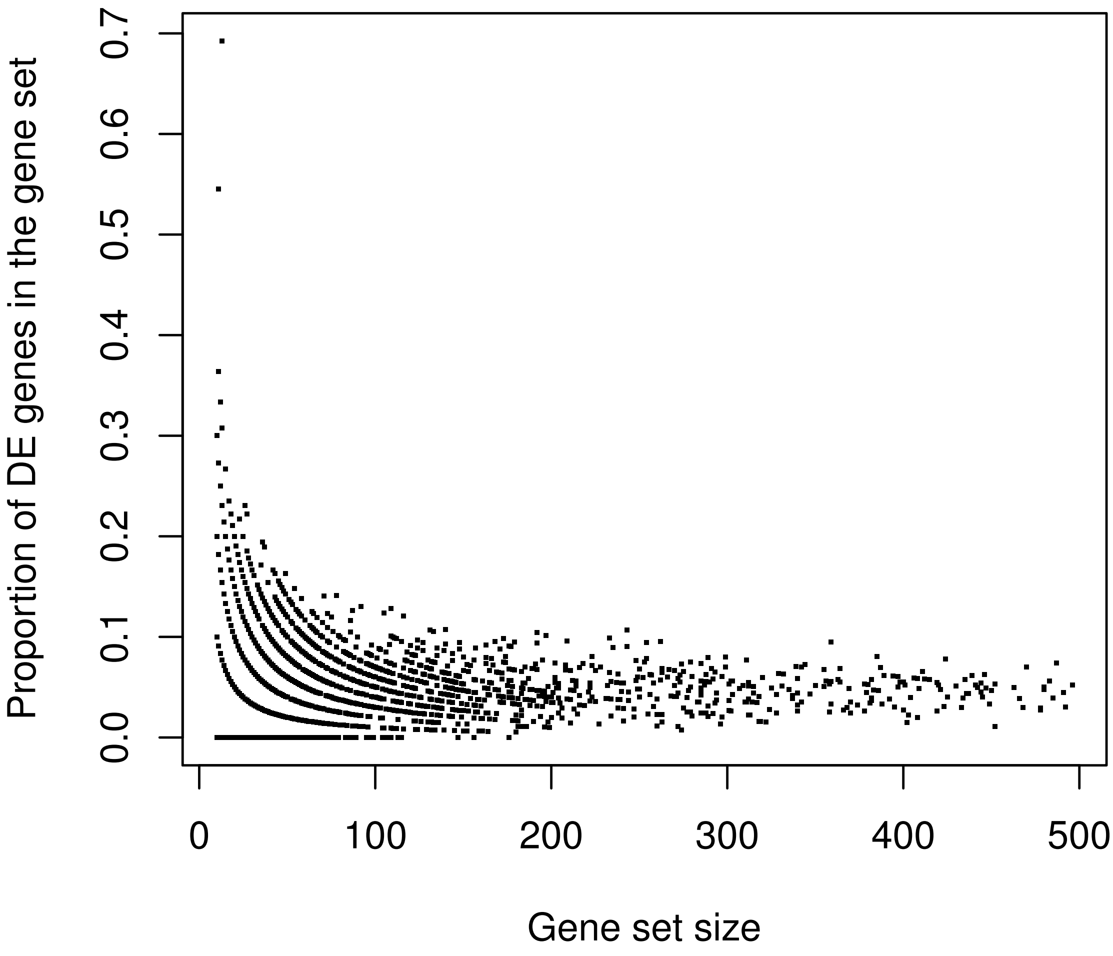

Figure S6: The proportion of differentially expressed (DE) genes found by eBayes in each of the 3890 C2 gene sets at a significance level $\alpha = 0.05$ versus gene set size for the Nigerian dataset.

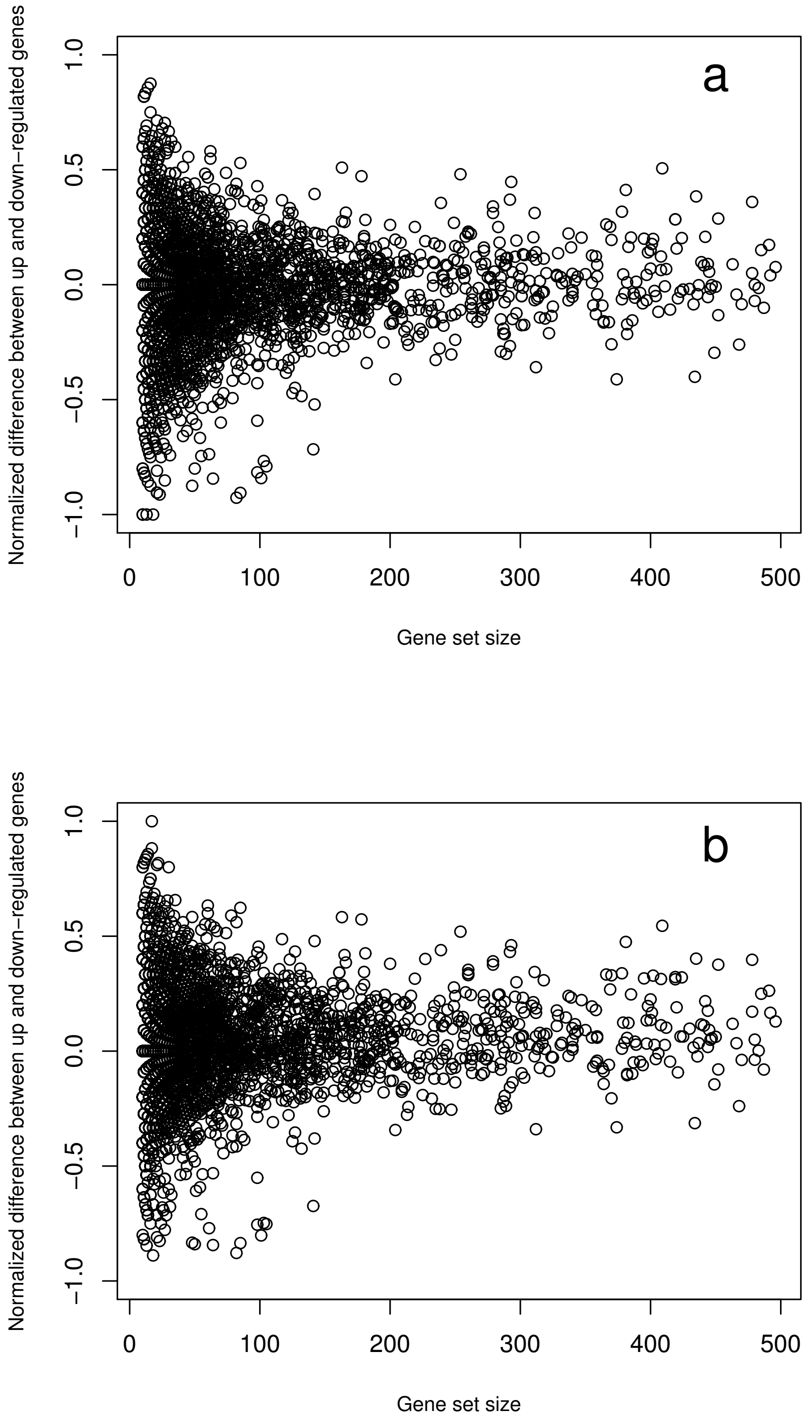

Figure S7: Difference between the number of up and down-regulated genes in each C2 gene set normalized by its size. (a) Using eBayes over VOOM-normalized data; (b) using edgeR over count data. Analyzing the Nigerian dataset in both panels show that small C2 gene sets have higher differences between the number of up and down-regulated genes than large C2 gene sets.
